# TRIM22 promotes the proliferation of glioblastoma cells by activating MAPK signaling and accelerating the degradation of Raf-1

**DOI:** 10.1038/s12276-023-01007-y

**Published:** 2023-06-01

**Authors:** Xiaowei Fei, Ya-nan Dou, Kai Sun, Jialiang Wei, Qingdong Guo, Li Wang, Xiuquan Wu, Weihao Lv, Xiaofan Jiang, Zhou Fei

**Affiliations:** 1 Department of Neurosurgery, Xijing Hospital, Air Force Military Medical University, Xi’an, Shaanxi China; 2 Department of Neurosurgery, Daping Hospital, Third Military Medical University, Chongqing, China

**Keywords:** CNS cancer, Ubiquitylation

## Abstract

The tripartite motif (TRIM) 22 and mitogen-activated protein kinase (MAPK) signaling pathways play critical roles in the growth of glioblastoma (GBM). However, the molecular mechanism underlying the relationship between TRIM22 and MAPK signaling remains unclear. Here, we found that TRIM22 binds to exon 2 of the sphingosine kinase 2 (*SPHK2*) gene. An ERK1/2-driven luciferase reporter construct identified TRIM22 as a potential activator of MAPK signaling. Knockout and overexpression of TRIM22 regulate the inhibition and activation of MAPK signaling through the RING-finger domain. TRIM22 binds to Raf-1, a negative regulator of MAPK signaling, and accelerates its degradation by inducing K48-linked ubiquitination, which is related to the CC and SPRY domains of TRIM22 and the C1D domain of Raf-1. In vitro and in vivo, an SPHK2 inhibitor (K145), an ERK1/2 inhibitor (selumetinib), and the nonphosphorylated mutant Raf-1^S338A^ inhibited GBM growth. In addition, deletion of the RING domain and the nuclear localization sequence of TRIM22 significantly inhibited TRIM22-induced proliferation of GBM cells in vivo and in vitro. In conclusion, our study showed that TRIM22 regulates *SPHK2* transcription and activates MAPK signaling through posttranslational modification of two critical regulators of MAPK signaling in GBM cells.

## Brain cancer: A protein and pathway to target glioblastoma

Key aspects of the role of the multi-functional protein TRIM22 in promoting the proliferation of glioblastoma, the most common malignant brain tumor in adults, have been identified in human glioblastoma cells and laboratory mice. Xiaofan Jiang, Zhou Fei and colleagues at the Air Force Medical University in Xi’an, China, revealed how TRIM22 activates MAPK signaling, a pathway of interacting proteins that controls cell growth and development. TRIM22 was also found to accelerate the degradation of a cell-regulating protein called Raf-1. This occurs when TRIM22 binds to and controls the expression of a gene involved in MAPK signaling, and also when it directly interacts with Raf-1 and another key protein. Inhibiting TRIM22 with a candidate drug suggested that targeting it or the processes it affects could yield new treatments for glioblastoma.

## Introduction

The mitogen-activated protein kinase (MAPK) cascade regulates a variety of cell biological processes, such as proliferation, differentiation, apoptosis, and key signaling pathways of the stress response under normal and pathological conditions^1–3^. Studies have shown that MAPK is highly active in human glioblastoma (GBM) and plays a central role in many other tumor-promoting pathways^4–6^. For example, the MAPK and PI3K signaling pathways cooperate to cause the pathogenesis of GBM^7^. However, mutation or amplification of MAPK signaling subunits is rare in GBM, which indicates that the abnormal activation of MAPK signaling may be due to the deregulation of pathways or oncogenes.

Ubiquitination is one of the most common and important types of posttranslational modification^8,9^. The most well-studied polyubiquitin chain types are lysine 48 (K48) and lysine 63 (K63). K48-linked polyubiquitin chains mainly target degraded proteins, whereas K63-linked polyubiquitin chains regulate kinase activity, signal transduction, and endocytosis^10,11^. Posttranslational modifications, especially ubiquitin modifications, are key processes underlying MAPK activation. For instance, LZTR1 promotes the polyubiquitination and degradation of Ras through the ubiquitin‒proteasome pathway, resulting in the inhibition of Ras/MAPK signaling^12^.

Tripartite motif (TRIM) proteins, which regulate MAPK activity through ubiquitination, are key regulators in the development of a variety of cancers, including GBM^9^. The TRIM-FLMN protein TRIM45 directly interacts with RACK1 and negatively regulates the PKC-mediated MAPK signaling pathway^13^. GPER decreases Bim protein expression through the MAPK/ERK-TRIM2 signaling axis and promotes the resistance of ER + breast cancer cells to tamoxifen^14^. The relationship of TRIM22 with the development of most human cancers has rarely been studied, and to date, the association between TRIM22 and the MAPK signaling pathway has not been reported. It has been reported that TRIM22 regulates protein degradation and activity as an E3 ubiquitin ligase in the cytoplasm^15^, but an increasing number of studies have found that TRIM22 is mainly located in the nucleus^16^. Using ChIP-Seq, CUT&Tag-qPCR, and CRISPR/Cas9 technologies, we found that TRIM22 acts as a transcription factor in the nucleus to regulate the expression of sphingosine kinase 2 (*SPHK2*) and then indirectly activates the MAPK pathway downstream of SPHK2. In addition, our study revealed that TRIM22, a classical E3 ubiquitin ligase, promotes K48-linked ubiquitination of Raf-1 and K63-linked ubiquitination of ERK1/2, thus directly affecting the MAPK pathway. Currently, SPHK2 and MAPK inhibitors have been widely used in clinical trials and have achieved good curative effects^17,18^. Our finding that TRIM22 directly and indirectly regulates the SPHK2/MAPK signaling pathway provides a new approach for GBM treatment.

## Materials and methods

### Ethics approval and consent to participate

All primary glioma tissue samples (WHO II, *n* = 92; WHO III, *n* = 65; WHO IV, *n* = 70) and nonneoplastic brain tissue samples (NBT, *n* = 10) were obtained from the Department of Neurosurgery at Xijing Hospital. The molecular profiles of WHO II samples were as follows (13 patients were not tested for molecular profile): IDH1/2 mutation (82.28%, 65/79), MGMT methylation (+, 56.96%, 45/79), P53 ( + 77.21%, 61/79), EGFR ( + 40.50%, 32/79), ARTX (−59.49%, 47/79). The molecular profiles of WHO III samples were as follows: IDH1/2 mutation (38.46%, 25/65), MGMT methylation (+49.23%, 32/65), P53 ( + , 66.15%, 43/65), EGFR ( + , 80%, 52/65), ARTX (-47.69%, 31/65). Molecular profiles of WHO IV samples: IDH1/2 mutation (28.57%, 20/70), MGMT methylation (+31.43%, 22/70), P53 ( + 68.57%, 48/70), EGFR ( + 88.57%, 62/70), ARTX (−42.86%, 30/70). Ethical approval was obtained from the Xijing Hospital Research Ethics Committee, and written informed consent was obtained from each patient. All experimental procedures were approved by the Institutional Animal Care and Use Committee of Fourth Military Medical University. This study was performed in accordance with the principles of the Declaration of Helsinki.

### Cell culture

Human glioma cell lines (U251MG, A172, U118, Hs683, SNB19, KNS89, H4, LN229, T98, U87MG, and TJ905) and 293 T cells were purchased from GeneChem Co., Ltd. (Shanghai, China). All cell lines were authenticated using short tandem repeat analysis. All cells were cultured in Dulbecco’s modified Eagle’s medium (DMEM; Gibco) containing 10% fetal bovine serum (FBS; Gibco).

Fresh clinical GBM samples obtained from the Department of Neurosurgery at Xijing Hospital were used to extract primary GBM cells using a stereomicroscope. The culture dish was coated with 0.2 mg/mL poly-L-lysine (Sigma‒Aldrich) and incubated overnight at 37 °C, washed three times with sterile water and placed in an incubator again until later use. The brain tissue was minced with sterile ophthalmic scissors and digested with 0.25% trypsin for 5 min at 37 °C before being centrifuged at 1000 rpm for 5 min. Serum-free neurobasal medium containing 2% B27 (Gibco) and 0.25% glutamine (Sigma) was used for primary cell culture. After two weeks of culture, primary cells adhered to the bottom of the culture dish. The culture medium was replaced by the Primary Cancer Culture System (PromoCell, Germany), and the cells were cultured for another week to gradually deplete the stromal cells (fibroblasts, epithelial cells, etc.) in the culture dish. Here, the primary cancer culture system was replaced with DMEM containing 10% FBS, and hTERT virus (pHBLV-CMV-hTERT-3FLAG-EF1-ZsGreen-T2A-PURO, Hanbio) was transfected into cells. The cells stably transfected with the virus were screened with puromycin and cultured for three weeks. GBM cells with unlimited proliferation capacity were retained. Finally, the morphological structure of the obtained primary cells was detected by scanning electron microscopy (SEM) and HE staining. The senescence level of the cells was detected using a senescence detection kit (Shanghai, China). Cells that grew well and were positive for GBM markers (GFAP, S100, Ki67, and CD133) were detected and screened by flow cytometry. According to the above method, we cultured GBM primary cells from a total of 13 patients and successfully developed two GBM cell lines with immortalization characteristics, named P1 and P2. The molecular profiles of P1 and P2 were as follows: IDH1/2 wild type, MGMT methylation (−), P53 ( + ), EGFR ( + ), ARTX ( + ). All cells were cultured in a constant temperature incubator containing 5% carbon dioxide at 37 °C.

### Lentivirus and plasmid transfection

*TRIM22* was knocked out using CRISPR/Cas9 technology. Cas9 and single guide RNA (sgRNA) lentiviruses were designed and constructed by GeneChem Co., Ltd. (Shanghai, China). The sequences of the sgRNAs used are listed in Supplementary Table 1. Cell lines were screened with puromycin.

*TRIM22* mutant plasmids, including Flag-TRIM22-Full length (FL), Flag-TRIM22-ΔRING, Flag-TRIM22-ΔB-Box, Flag-TRIM22-ΔCC and Flag-TRIM22-ΔSPRY, and Raf-1 mutant plasmids, including HA-Raf-1-FL, HA-Raf-1-ΔRBD, HA-Raf-1-C1D and HA-Raf-1-CD, were designed and constructed by Hanbio Co., Ltd. (Shanghai, China). Plasmid transfection was performed using jetPRIME DNA transfection reagent (PolyPlus) according to the manufacturer’s instructions.

The nuclear localization sequence (NLS) of TRIM22 is in the 257-275 aa region. The NLS deletion plasmid Flag-TRIM22-ΔNLS was designed and constructed by Hanbio Co. Ltd. (Shanghai, China).

### Subcellular fractionation

Nuclear and cytoplasmic fractions from U251MG, A172, TJ905, H4, P1, and P2 cells were isolated using the Nuclear and Cytoplasmic Extraction Kit purchased from Beyotime (Shanghai, China) according to the manufacturer’s instructions. The subcellular distribution of proteins was determined using Western blot analysis. GAPDH and histone H3 served as loading controls for the cytosolic and nuclear fractions, respectively.

### Cleavage under targets and tagmentation (CUT&Tag)

CUT&Tag was carried out according to the instructions of the Hyperactive kit, The In Situ ChIP Library Prep Kit for Illumina (pG-Tn5) (Cat. No. TD901-01), VAHTS DNA Clean Beads (Cat. No. N411), TruePrep Index Kit V2 (Cat. No. TD202) and Equalbit 1 × dsDNA HS Assay Kit (Cat. No. EQ121). All kits were purchased from Vazyme (Nanjing, China).

### Quantitative real-time polymerase chain reaction (qPCR)

qPCR was performed according to the protocol of the ChamQ SYBR Color qPCR Master Mix (Low ROX Premixed) kit (Vazyme, China). The RNA expression level of genes of interest was calculated by the 2^−ΔΔCt^ method, and GAPDH served as an internal control. The primer sequences used for ChIP‒qPCR are listed in Supplementary Table 2.

### Chromatin immunoprecipitation sequencing and qPCR (ChIP-Seq and ChIP‒qPCR)

ChIP-Seq and ChIP‒qPCR were performed with assistance from Kangchen Biotechnology Co., Ltd. (Shanghai, China). The detailed steps of the experiment are described in the Supplementary Materials and Methods. The efficiency of coimmunoprecipitation (ChIP/Total input) of the gene to be tested was calculated by the following formula: % (ChIP/Total input) = 2 ^ [Ct (Input) - Ct (ChIP)] *100.

### Dual-luciferase reporter assay

ERK1/2 firefly luciferase and Renilla reporter plasmids were transfected into each modified cell line. The cells were cultured for 24 h after plasmid transfection, and the fluorescence intensity of each treatment group was detected using the Dual-Luciferase Reporter Assay Kit (Promega).

### Immunofluorescence (IF), immunohistochemistry (IHC), coimmunoprecipitation (Co-IP) and Western blotting (WB)

IF, IHC, and WB were performed as previously described,^19,20^ and all antibodies and reagents used are described in Supplementary Table 4. To assess in vivo ubiquitination, modified cells were treated with 20 μM MG132 (Apexbio, Houston, TX, USA) for 12 h before lysis, followed by Western blot analysis and Co-IP.

### Yeast one-hybrid assay

Different modified potential DNA-binding proteins, the prey, were expressed as fusions to the GAL4 activation domain in pGADT7-Rec2. The different modified target DNA sequences, or bait sequences, were each cloned into the pHIS2.1 reporter vector as three tandem repeats. The two plasmids were transferred into the yeast strain Y187 and cultured in the appropriate media. 3-Amino-1,2,4-triazole (3-AT) was used to inhibit low-level His3p expression in the absence of an activating prey protein.

### Animal studies

Four-week-old male NOD/SCID and C57BL/6 mice were purchased from Shenzhen Huafukang Bioscience Co., Inc. (Shenzhen, China) and were randomly divided into groups of 5 mice each. Different modified GBM cells expressing luciferase were implanted into the brains of mice by stereotactic injection (2 mm posterior and 1 mm lateral to the bregma). Tumor cells (3 × 10^6^/5 μL PBS) were injected into the mouse brain (depth: 2.8 mm) using a microsyringe within 2 min. After the operation, the skull was sealed with bone wax, the incision was sutured, and glucose (0.5 mL) was injected into the abdominal cavity. Fluorescence bioluminescence detection equipment (IVIS Lumina III) was used to detect tumor formation 7, 15, and 30 days after surgery. K145 (50 mg/kg) and selumetinib (50 mg/kg) were administered intraperitoneally every day until the end of the experiment according to the weights of the mice.

Mortality and tumorigenicity were assessed throughout the experiment, the endpoint was selected to ensure that there were at least three mice that could be tested for indicators.

### Staining of cell clones

A172 and P1 cells were counted separately, and 5000 cells each were cultured in 10-mm dishes at the optimal density. After complete attachment, cells were transfected with plasmids containing different *TRIM22* mutants according to the experimental groups. The medium was replaced with fresh medium after 24 h. After an additional two weeks of incubation, staining was performed with 1% crystal violet dye for 15 min, and random fields were selected for cell counting and quantification under a microscope after photography.

### Cell viability assay

Cell viability was assessed using the CCK-8 assay. After the cells were modified with different *TRIM22* mutant plasmids, they were seeded at a density of 2000 cells/well in 96-well plates and incubated for 48 h. Ten microliters of CCK-8 reagent was added to each well and incubated for 1 h at 37 °C. The optical density (OD) value was read at 450 nm using Thermo Fisher Scientific SkanIt Software 3.1 for Multiskan GO. The viability rate of cells = (OD values of treated groups/the OD values of the control group) × 100%.

### Statistical analysis

All experiments were performed in at least three independent biological replicates. Data are expressed as the mean ± standard deviation. A power analysis was performed to ensure a proper sample size. Student’s t test was used for statistical analysis between two groups, and one-way analysis of variance (ANOVA) was used to compare multiple groups. The F test was used to check whether the data conformed to a normal distribution. Prism 9 for macOS was used for statistical analyses. The threshold for statistical significance was set at *P* < 0.05.

## Results

### TRIM22 acts as a transcription factor to regulate the transcription of *SPHK2* in the nucleus

**Fig. 1:**
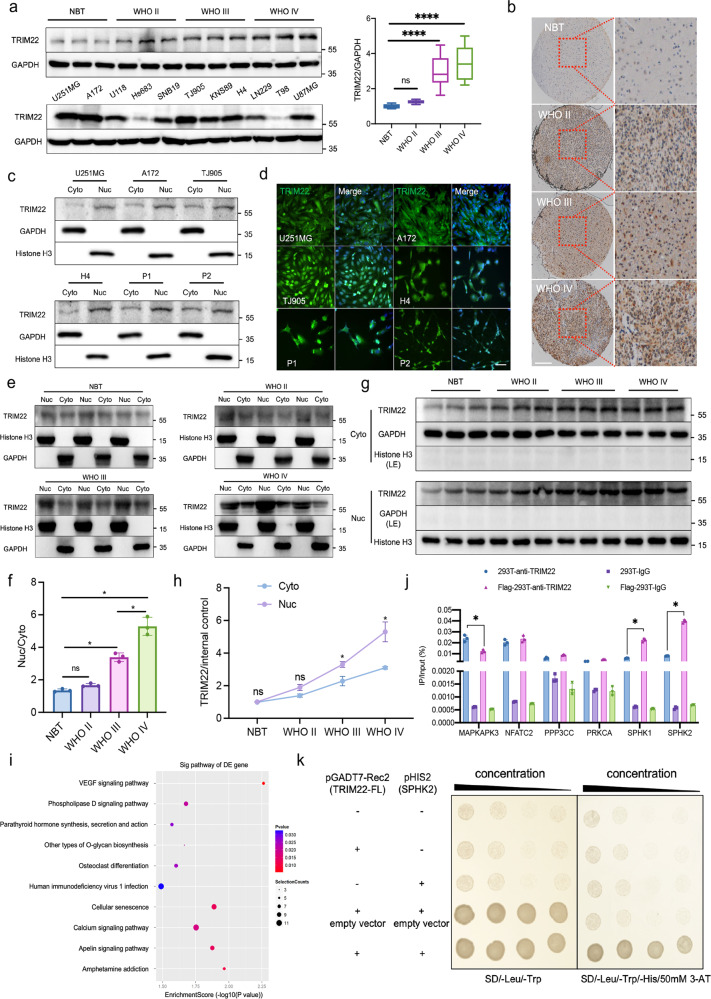
TRIM22 regulates the transcription of *SPHK2* as a transcription factor in the nucleus. **a** Representative Western blot images and quantification of TRIM22 expression in different grades of gliomas (upper) and TRIM22 expression in 11 different GBM cell lines was assessed by Western blotting (lower). F (3, 233) = 188.2, *P* < 0.0001. **b** The localization of TRIM22 in clinical specimens was detected by immunohistochemistry. Scale bar: 200 μm. Number of clinical samples in **a** and **b**: NBT; *n* = 10, WHO II; *n* = 92, WHO III; *n* = 65, WHO IV; *n* = 70. **c** Expression of TRIM22 in the cytoplasm and nucleus of U251MG, A172, TJ905, H4 and primary GBM cells P1 and P2. **d** Localization of TRIM22 in six cell lines. Scale bar: 50 μm. **e** Representative Western blot images of TRIM22 expression in the nucleus (Nuc) and cytoplasm (Cyto) of gliomas of different grades (on different gels). **f** Quantification of the results in Panel e (*n* = 3 per group). F (3, 8) = 99.44, *P* < 0.0001. **g** Representative Western blot images of TRIM22 expression in the nucleus (Nuc) and cytoplasm (Cyto) of gliomas of different grades (on one gel). **h** Quantification of the results in Panel g (*n* = 3 per group). F_interaction_(3, 16) = 19.10, *P* < 0.0001. **i** After 293 T cells were transfected with the Flag-TRIM22 overexpression plasmid, ChIP-Seq and KEGG analyses were carried out to analyze the differentially expressed genes. **j** ChIP‒qPCR was used to verify the results of ChIP-Seq (*n* = 3 per group). MAPKAPK3: F (3, 8) = 133.4, *P* < 0.0001; NFATC2: F (3, 8) = 116.5, *P* < 0.0001; PPP3CC: F (3, 8) = 222.8, *P* < 0.0001; PPKCA: F (3, 8) = 385.6, *P* < 0.0001; SPHK1: F (3, 8) = 547.1, *P* < 0.0001; SPHK2: F (3, 8) = 1896, *P* < 0.0001. **k** A yeast one-hybrid experiment verified the binding of TRIM22 and *SPHK2* in vitro. The data were analyzed using one-way **a**, **f**, and **j** or two-way analysis **h** of variance, and all data are expressed as the mean ± standard deviation. *P < 0.05 and ****P < 0.0001 represent a statistically significant difference between the two groups. ns, not significant. Each experiment was repeated three times.

We collected glioma samples of different grades from Xijing Hospital for detection (NBT, *n* = 10; WHO II, *n* = 92; WHO III, *n* = 65; WHO IV, *n* = 70). We found that TRIM22 was highly expressed in GBM cells (Fig. 1a) (F (3, 233) = 188.2, *P* < 0.001) and was mainly located in the nucleus (Fig. 1b). In addition, the cell lines U251MG, A172, TJ905, and H4 with high TRIM22 expression and the primary GBM cell lines P1 and P2 were selected. Immortalized P1 and P2 cells that were positive for the markers GFAP, S100, Ki67, and CD133 and survived well were selected by flow cytometry (Supplementary Fig. 1a). We found that there was no senescence in P1 and P2 cells (Supplementary Fig. 1b). In addition, the results of HE staining (Supplementary Fig. 1c) and SEM (Supplementary Fig. 1d) showed that P1 and P2 cells had disproportionate nucleoplasm, hyperchromatic nuclei, coarse chromatin, and high mitotic figure count. These results illustrate that primary GBM cells P1 and P2 were successfully extracted and immortalized. Western blotting (Fig. 1c) and immunofluorescence (Fig. 1d) showed that TRIM22 was mainly localized in the nucleus. Compared with that in the nuclei of low-grade gliomas, TRIM22 expression was greater in the nuclei of high-grade gliomas (Figs. 1e, 1f). Although the expression level of TRIM22 in the nucleus and cytoplasm was positively correlated with the grade of glioma, the correlation was stronger for nuclear TRIM22 expression (Fig. 1g, h). To study the role of TRIM22 in the nucleus, we used ChIP-Seq and enrichment analysis and found that TRIM22 binds to DNA fragments of VEGF pathway-related genes (*MAPKAPK3*, *NFATC2*, *PP3CC*, *PRKCA*, *SPHK1*, and *SPHK2*), which may play a role as transcription factors (Fig. 1i, Supplementary Fig. 2a). The results of ChIP‒qPCR showed that the input-IP/input-negative values of the six genes were 0.552, 1.288, 2.135, 1.98, 2.109, and 4.062, respectively. Input-IP/input-negative ratios greater than three were considered reliable. In addition, ChIP‒qPCR (Fig. 1j) in 293 T cells and the yeast one-hybrid assay (Fig. 1k) showed that TRIM22 could bind to a fragment of the *SPHK2* gene in vivo. These results show that TRIM22 acts as a transcription factor that regulates the transcription of *SPHK2* in the nucleus.

### TRIM22 binds to the transcription site of *SPHK2*

**Fig. 2:**
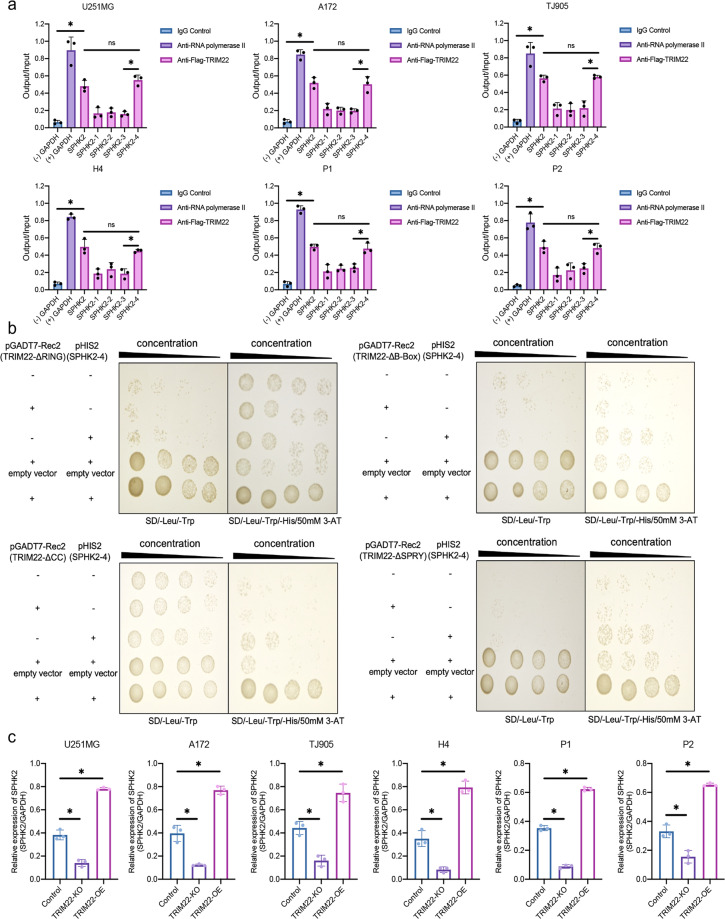
TRIM22 binds to the transcription site of *SPHK2* and positively regulates its transcription. **a** Different binding sites of TRIM22 and *SPHK2 (1-4)* were detected by CUT&Tag in U251MG [F (6, 14) = 47.44, *P* < 0.0001], A172 [F (6, 14) = 70.91, *P* < 0.0001], TJ905 [F (6, 14) = 46.58, *P* < 0.0001], H4 [F (6, 14) = 68.34, *P* < 0.0001] and primary P1 [F (6, 14) = 98.16, *P* < 0.0001] and P2 [F (6, 14) = 36.82, *P* < 0.0001] GBM cells. *n* = 3 per group. **b** In vitro yeast one-hybrid assays were used to detect the binding of different TRIM22 truncation mutants (Flag-TRIM22-ΔRING, Flag-TRIM22-ΔB-Box, Flag-TRIM22-ΔCC and Flag-TRIM22-ΔSPRY) to *SPHK2-4*. **c** Effect of knockout or overexpression of *TRIM22* on SPHK2 mRNA expression in U251MG [F (2, 6) = 325.3, *P* < 0.0001], A172 [F (2, 6) = 153.6, *P* < 0.0001], TJ905 [F (2, 6) = 68.89, *P* < 0.0001], H4 [F (2, 6) = 138.5, *P* < 0.0001] and primary P1 [F (2, 6) = 1047, *P* < 0.0001] and P2 [F (2, 6) = 144.7, *P* < 0.0001] GBM cells. *n* = 3 per group. The data were analyzed using one-way analysis of variance, and all data are expressed as the mean ± standard deviation. *P < 0.05 represents a statistically significant difference between the two groups. ns, not significant. Each experiment was repeated three times.

According to information on multiple transcripts of *SPHK2*, the transcription start site (TSS), and the position of the peak in the sequencing results, four pairs of different primers were designed to explore potential binding sites between TRIM22 and *SPHK2* (Supplementary Table 3, Supplementary Fig. 2b). In 293 T cells, ChIP‒qPCR showed that TRIM22 was most closely bound to the peak region (Supplementary Fig. 2c). In addition, different truncated forms of TRIM22 (Flag-TRIM22-ΔRING, Flag-TRIM22-ΔB-Box, Flag-TRIM22-ΔCC, and Flag-TRIM22-ΔSPRY) were transfected into U251MG, A172, TJ905, H4, and two primary cell lines, P1 and P2, for CUT&Tag. Surprisingly, both TRIM22 (Fig. 2a) and its truncated forms (Supplementary Fig. 3a, b, Supplementary Fig. 4a, b) bound to *SPHK2-4*. In the yeast one-hybrid assay, three repeated sequences for the *SPHK2-4* genes were used as bait sequences, and we obtained results consistent with those in vivo (Fig. 2b). To further explore how TRIM22 regulates *SPHK2*, we constructed a *TRIM22*-knockout (KO) cell line using CRISPR/Cas9 technology (Supplementary Fig. 2d). The qPCR results showed that knockout of *TRIM22* reduced the mRNA expression level of *SPHK2*, whereas overexpression of TRIM22 promoted its expression in four cell lines and two primary cell lines (Fig. 2c). These results suggested that TRIM22 positively regulates the expression of *SPHK2*.

### TRIM22 positively regulates the SPHK2/MAPK signaling pathway through K48-linked ubiquitination of Raf-1

**Fig. 3:**
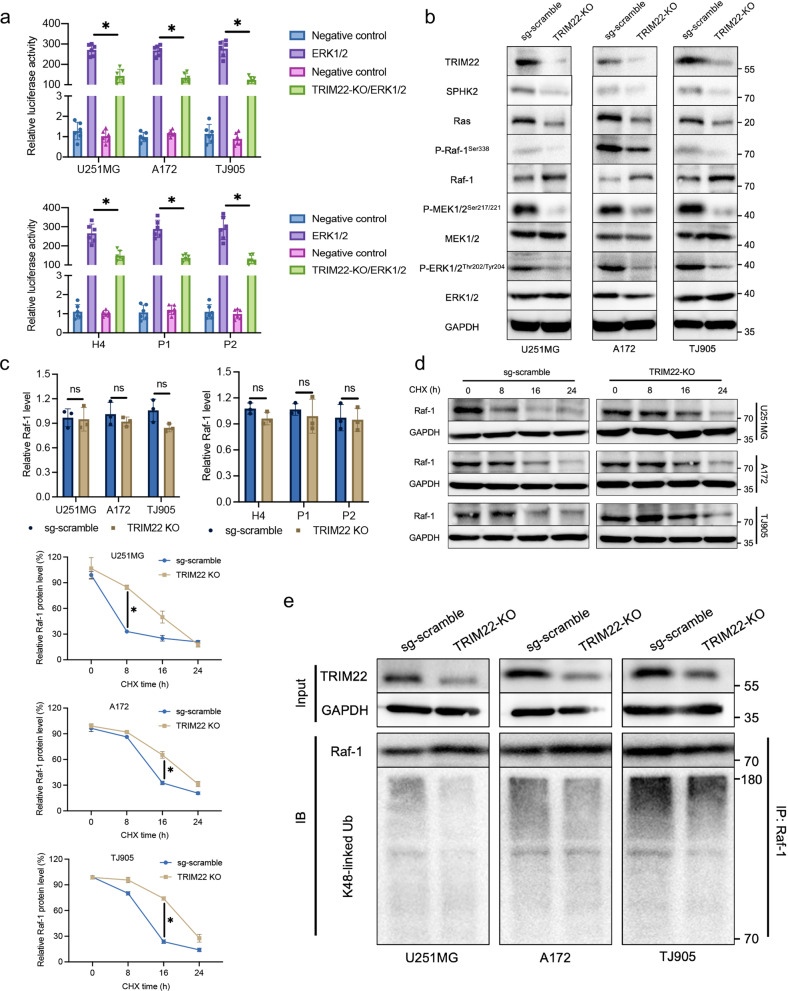
*TRIM22* deletion inhibits SPHK2/MAPK signaling via K48-linked ubiquitination of Raf-1. **a** Luciferase activity in U251MG [F (3, 20) = 219.2, *P* < 0.0001], A172 [F (3, 20) = 336.7, *P* < 0.0001], TJ905 [F (3, 20) = 220.5, *P* < 0.0001], H4 [F (3, 20) = 128.5, *P* < 0.0001] and primary P1 [F (3, 20) = 190.8, *P* < 0.0001] and P2 [F (3, 20) = 111.1, *P* < 0.0001] GBM cells transfected with sgRNA-TRIM22, along with a reporter plasmid carrying the ERK1/2 promoter relative to negative control. Relative fluorescence intensity: Firefly luciferase/Renilla luciferase. *n* = 6 per group. **b** The expression of SPHK2/MAPK pathway core proteins was detected by Western blotting. **c** The mRNA level of Raf-1 was detected by qPCR (*n* = 3 per group. U251MG: t = 0.1778, df = 4, *P* = 0.8675; A172: t = 1.1002, df = 4, *P* = 0.3647; TJ905: t = 2.542, df = 4, *P* = 0.0639; H4: t = 2.056, df = 4, P = 0.109; P1: t = 0.642, df = 4, *P* = 0.0.5558; P2: t = 0.2058, df = 4, *P* = 0.847; **d** Western blot analysis and quantification of Raf-1 protein in *TRIM22-KO* cells treated with cycloheximide (CHX; 25 μg/mL) for 0, 8, 16, and 24 h (*n* = 3 per group). U251MG: F_interaction_(3, 16) = 26.99, *P* < 0.0001; A172: F_interaction_(3, 16) = 40.20, *P* < 0.0001; TJ905: F_interaction_(3, 16) = 106.4, *P* < 0.0001. **e** IP experiments were used to detect the endogenous K48-linked ubiquitination of Raf-1 after *TRIM22* knockout. The data were analyzed using one-way ANOVA **a**, Student’s t test **c** or two-way ANOVA **d**, and all data are expressed as the mean ± standard deviation. *P < 0.05 represents a statistically significant difference between the two groups. ns, not significant. Each experiment was repeated three times.

Our results showed that TRIM22 regulates the transcription of *SPHK2* and positively regulates SPHK2 expression. The enrichment results from the ChIP-Seq experiment also showed that TRIM22 may affect the VEGF pathway; although both the MAPK and PI3K/AKT pathways are involved in the VEGF pathway, only the MAPK pathway is downstream of SPHK2 (Supplementary Fig. 2a). To explore the effect of TRIM22 on the SPHK2/MAPK pathway, we used an ERK1/2-dependent transcriptional reporter containing three copies of an ERK1/2 response element located upstream of luciferase. The dual-luciferase reporter assay showed that knockout of *TRIM22* inhibited MAPK signaling (Fig. 3a). Knockout of *TRIM22* inhibited the expression of SPHK2/MAPK pathway proteins, including SPHK2, Ras, P-Raf-1^Ser338^, P-MEK1/2^Ser217/221^ and P-ERK1/2^Thr202/Tyr204^. Although the phosphorylation of MEK1/2 and ERK1/2 was suppressed after knockout of *TRIM22*, there was no significant change in the expression of MEK1/2 and ERK1/2 (Fig. 3b, Supplementary Fig. 5a). Interestingly, the protein level of Raf-1 was increased, but its mRNA level did not change (Fig. 3c). In addition, knockout of *TRIM22* attenuated the degradation of Raf-1 (Fig. 3d, Supplementary Fig. 5b) and reduced K48-linked ubiquitination of Raf-1 (Fig. 3e, Supplementary Fig. 5c). These results suggest that TRIM22 regulates the SPHK2/MAPK pathway and that *TRIM22* deletion attenuates the K48-linked ubiquitination of Raf-1.

### SPHK2 and MAPK inhibitors attenuated TRIM22-promoted proliferation of GBM

**Fig. 4:**
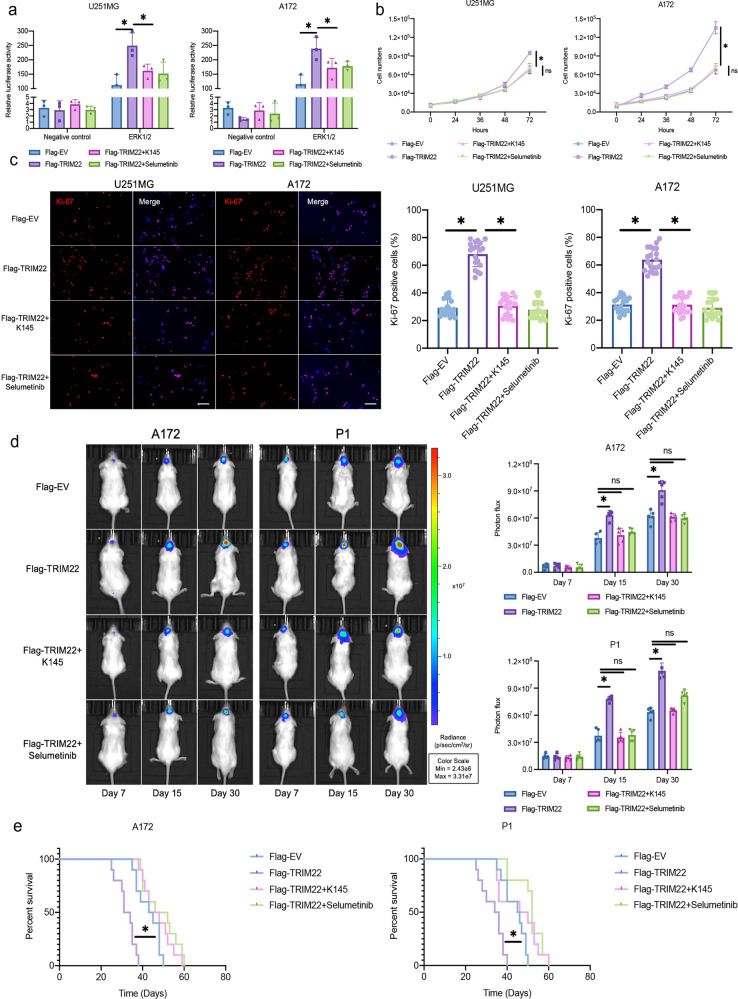
The SPHK2/MAPK pathway regulates the proliferation of GBM in vivo and in vitro. **a** Luciferase activity from U251MG and A172 cells treated with K145 and selumetinib, along with a reporter plasmid carrying the ERK1/2 promoter relative to the negative control. Relative fluorescence intensity: Firefly luciferase/Renilla luciferase (*n* = 3 per group). U251MG: F_interaction_(3, 16) = 7.32, *P* = 0.0026; A172: F_interaction_(3, 16) = 7.828, *P* = 0.0019. **b** Growth curves generated using cell counting data over 72 h for the indicated cells (*n* = 3 per group). U251MG: F_interaction_(12, 40) = 8.438, *P* < 0.0001; A172: F_interaction_(12, 40) = 35.17, *P* < 0.0001. **c** Representative images and quantification of Ki-67 immunofluorescence staining from modified U251MG [F (3, 76) = 150.8, *P* < 0.0001] and A172 cells [F (3, 76) = 118.5, *P* < 0.0001] (*n* = 20 per group). Scale bar: 50 μm. Blue: DAPI. **d** Representative images and quantification of in vivo imaging (*n* = 5 mice per group). A172: F_interaction_(6, 48) = 9.857, *P* < 0.0001; P1: F_interaction_(6, 48) = 29.75, *P* < 0.0001. **e** Kaplan–Meier survival analysis and log-rank test performed with survival data from the indicated groups (*n* = 10 mice per group). A172: Log-rank (Mantel‒Cox) test, Chi square = 48.88, df = 3, *P* < 0.0001; P1: Log-rank (Mantel‒Cox) test, Chi square = 31.87, df = 3, *P* < 0.0001. In the in vitro experiment, cells were treated with 5 µM K145 or 10 nM selumetinib for 24 hours. The data were analyzed using one-way ANOVA **c** or two-way ANOVA (**a**, **b** and **d**), and all data are expressed as the mean ± standard deviation. *P < 0.05 represents a statistically significant difference between the two groups. ns, not significant. Each experiment was repeated three times. EV: empty vector.

It has been reported that TRIM22 is highly expressed in GBM and promotes the proliferation of GBM^21^. To explore whether TRIM22 promotes GBM cell proliferation through the SPHK2/MAPK signaling pathway, K145, an SPHK2 inhibitor, and selumetinib, a highly potent non-ATP-competitive MEK1/2 inhibitor, were used for in vivo and in vitro experiments. Overexpression of TRIM22 activated MAPK signaling, whereas the fluorescence intensity decreased significantly after treatment with K145 and selumetinib for 24 h (Fig. 4a, Supplementary Fig. 6a). K145 and selumetinib also inhibited the growth of cells promoted by TRIM22 (Fig. 4b, Supplementary Fig. 6b) and decreased the expression rate of Ki67 (Fig. 4c, Supplementary Fig. 6c). Consistent with previous reports, the A172 GBM cell line and the primary GBM cell line P1, both of which exhibit TRIM22 overexpression, showed accelerated proliferation 15 days after tumor implantation. The tumor growth rate of NOD/SCID mice intraperitoneally injected daily with K145 and selumetinib was slower (Fig. 4d). In addition, injection of K145 and selumetinib increased the survival rate of mice (A172: Flag-EV vs. Flag-TRIM22; 44 d vs. 32.5 d; *P* < 0.05; Flag-TRIM22 vs. Flag-TRIM22 + K145 or Flag-TRIM22 + selumetinib; 32.5 d vs. 45.5 or 49 days; *P* < 0.05; P1: Flag-EV vs. Flag-TRIM22; 45.5 d vs. 35 d; *P* < 0.05; Flag-TRIM22 vs. Flag-TRIM22 + K145 or Flag-TRIM22 + selumetinib; 35 d vs. 48 or 52 d; *P* < 0.05; Fig. 4e). These results illustrate that TRIM22 promotes GBM cell proliferation through the SPHK2/MAPK signaling pathway.

### TRIM22 ubiquitinates Raf-1

**Fig. 5:**
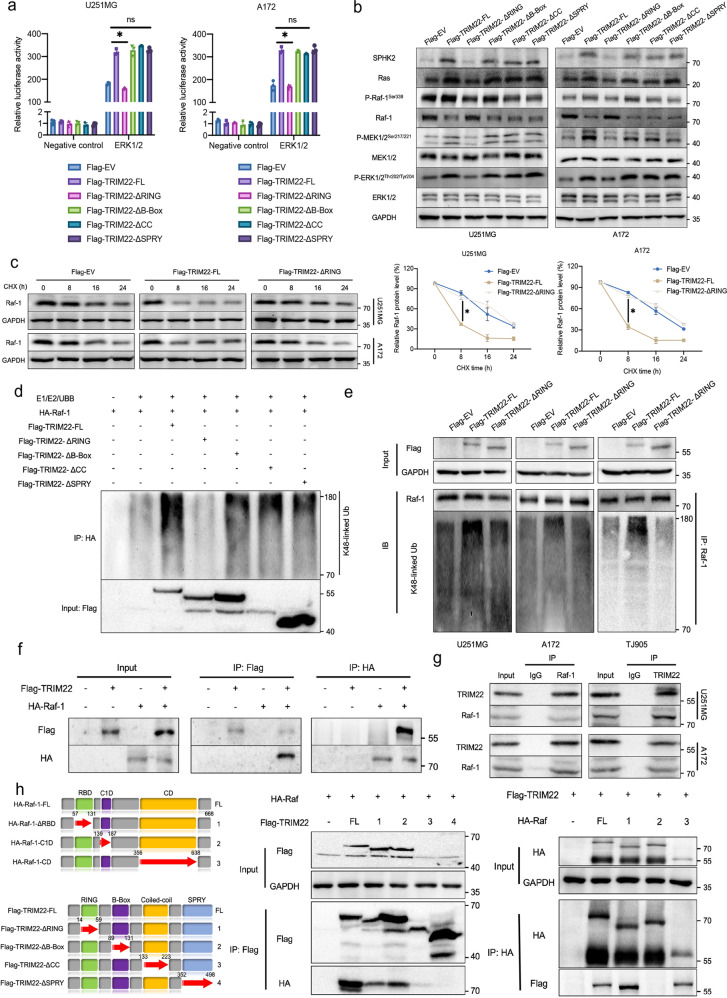
TRIM22 binds to Raf-1 and regulates SPHK2/MAPK signaling through its RING domain. **a** Luciferase activity in U251MG and A172 cells transfected with different TRIM22 truncation mutants (*n* = 3 per group). U251MG: F_interaction_(5, 24) = 145.2, *P* < 0.0001; A172: F_interaction_(5, 24) = 113.2, *P* < 0.0001. **b** The effects of different TRIM22 truncation mutants on the core protein of the SPHK2/MAPK pathway were detected by Western blotting in U251MG and A172 cells. **c** Western blot analysis and quantification of Raf-1 protein in different modified cells treated with cycloheximide (CHX; 25 μg/mL) for 0, 8, 16, and 24 h (*n* = 3 per group). U251MG: F_interaction_(6, 24) = 30.31, *P* < 0.0001; A172: F_interaction_(6, 24) = 75.78, P < 0.0001. **d** Ubiquitination of Raf-1 in an in vitro assay. **e** Ubiquitination assay of Raf-1 in modified U251MG, A172 and TJ905 cells. **f** Co-IP was used to detect the exogenous binding of TRIM22 and Raf-1 in 293 T cells transfected with Flag-TRIM22 and HA-Raf-1. **g** Exogenous binding of TRIM22 and Raf-1 in U251MG and A172 cells using anti-TRIM22 and anti-Raf-1 antibodies. **h** Schematic representation of wild-type TRIM22 and Raf-1 and the indicated deletion mutants. Western blot analysis of Co-IPs in 293 T cells transfected with Flag-TRIM22/HA-Raf-1 alone or together with the indicated HA-Raf-1/Flag-TRIM22 constructs. The data were analyzed using two-way ANOVA, and all data are expressed as the mean ± standard deviation. *P < 0.05 represents a statistically significant difference between the two groups. ns, not significant. Each experiment was repeated three times. EV: empty vector.

TRIM22 contains four domains: RING, B-Box, CC, and SPRY. To determine which is critical for TRIM22-mediated activation of MAPK signaling, we used constructs lacking these domains (TRIM22-ΔRING, TRIM22-ΔB-Box, TRIM22-ΔCC and TRIM22-ΔSPRY) in ERK1/2 luciferase reporter assays and compared these groups with the TRIM22-FL and EV groups. Compared with the TRIM22-FL group, only the TRIM22-ΔRING group showed significantly reduced luciferase activity (Fig. 5a, Supplementary Fig. 7a). Furthermore, TRIM22 overexpression increased the expression of key proteins in the SPHK2/MAPK pathway, while the lack of the RING domain inhibited this change. The change trend of Raf-1 protein was opposite to that of the other proteins (Fig. 5b, Supplementary Fig. 7b). In the cycloheximide (CHX) assay, TRIM22 overexpression accelerated the degradation of Raf-1, and the results were similar in the TRIM22-ΔRING and Flag-EV groups (Fig. 5c, Supplementary Fig. 7c). In both the in vitro (Fig. 5d) and cultured cells (Fig. 5e, Supplementary Fig. 7d), TRIM22 overexpression induced endogenous K48-linked ubiquitination of Raf-1, whereas RING domain deletion had no effect. These results illustrate that the change in Raf-1 expression is caused by the ubiquitin-dependent degradation pathway induced by TRIM22.

To investigate whether TRIM22 directly binds to Raf-1, we performed co-IP experiments using 293 T cells transfected with Flag-TRIM22 and HA-Raf-1, and Flag and HA were both detected, indicating that the proteins associate with each other (Fig. 5f). Furthermore, the physical binding of endogenous TRIM22 and Raf-1 was confirmed in four cell lines and two primary cell lines (Fig. 5g, Supplementary Fig. 7e). To further study which domain of the two proteins plays a crucial role in binding, a series of TRIM22 and Raf-1 deletion mutants were constructed and transfected into 293 T cells. The CC and SPRY domains of TRIM22 were necessary for reducing HA-Raf-1 expression, while the C1d domain of Raf-1 was necessary for reducing Flag-TRIM22 expression (Fig. 5h). Thus, TRIM22 promotes the proteasome-mediated degradation of Raf-1 via its RING domain and is a negative regulator of MAPK signaling.

### Raf-1 mediates the proliferation of GBM cells promoted by TRIM22

**Fig. 6:**
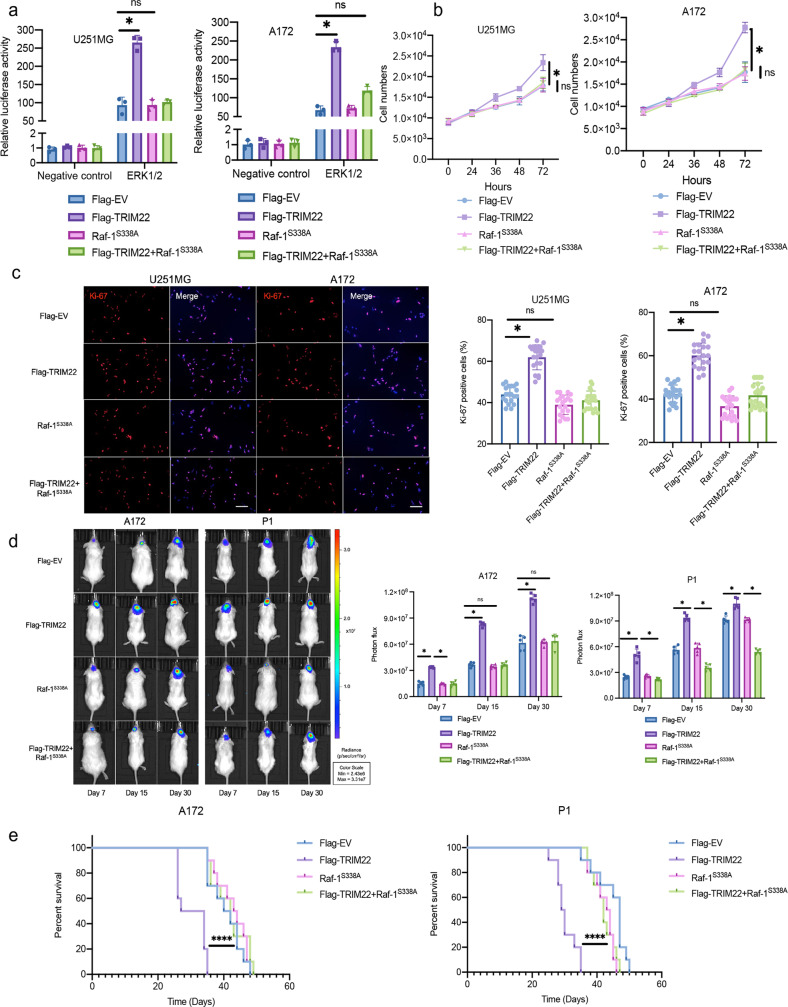
Raf-1 regulates TRIM22-induced GBM cell proliferation in vivo and in vitro. **a** Luciferase activity of U251MG and A172 cells transfected with Flag-TRIM22 and Raf-1^S338A^, along with a reporter plasmid carrying the ERK1/2 promoter relative to the negative control (*n* = 3 per group). U251MG: F_interaction_(3, 16) = 75.25, *P* < 0.0001; A172: F_interaction_(3, 16) = 145.6, *P* < 0.0001. **b** Growth curves generated using cell counting data over 72 h for the indicated cells (*n* = 3 per group). U251MG: F_interaction_(12, 40) = 5.665, *P* < 0.0001; A172: F_interaction_(12, 40) = 16.35, P < 0.0001. **c** Representative images and quantification of Ki-67 immunofluorescence staining from modified U251MG and A172 cells (*n* = 20 per group). Scale bar: 50 μm. Blue: DAPI. U251MG [F (3, 76) = 91.93, P < 0.0001] and A172 cells [F (3, 76) = 80.77, P < 0.0001]. **d** Representative images and quantification of in vivo imaging (*n* = 5 mice per group). A172: F_interaction_(6, 48) = 21.26, *P* < 0.0001; P1: F_interaction_(6, 48) = 23.33, *P* < 0.0001. **e** Kaplan–Meier survival analysis and log-rank test performed with survival data from the indicated groups (*n* = 10 mice per group). A172: Log-rank (Mantel‒Cox) test, Chi square = 42.80, df = 3, *P* < 0.0001; P1: Log-rank (Mantel‒Cox) test, Chi square = 55.71, df = 3, *P* < 0.0001. The data were analyzed using one-way ANOVA (**c**) or two-way ANOVA (**a,** **b** and **d**), and all data are expressed as the mean ± standard deviation. *P < 0.05 indicates a statistically significant difference between the two groups. ns, not significant. Each experiment was repeated three times.

Phosphorylation at S338 activates the Raf-1 protein^22^. To further study the role of Raf-1 in TRIM22-promoted proliferation of GBM, we constructed a mutant of phosphorylated Raf-1 in which the serine at site 338 was mutated to alanine (Raf-1^S338A^). The plasmid was packaged with lentivirus and transfected into the cells for stable expression. The luciferase reporter assay showed that Raf-1^S338A^ inhibited the activation of the MAPK pathway caused by TRIM22 overexpression (Fig. 6a, Supplementary Fig. 8a). The increases in the growth of GBM cells (Fig. 6b, Supplementary Fig. 8b) and positive expression rate of Ki67 (Fig. 6c, Supplementary Fig. 8c) induced by TRIM22 overexpression were also attenuated by Raf-1^S338A^. Moreover, in vivo, compared with the Flag-TRIM22 group, the Flag-TRIM22 + Raf-1^S338A^ group showed a significant inhibitory effect 7 days after intracranial tumor implantation (Fig. 6d). The Flag-TRIM22 group had shorter survival, whereas there was no difference between the Flag-EV, Raf-1^S338A^, and Flag-TRIM22 + Raf-1^S338A^ groups (A172, Flag-EV vs. Flag-TRIM22 vs. Raf-1^S338A^ vs. Flag-TRIM22 + Raf-1^S338A^: 41 d vs. 30.5 d vs. 43.5 d vs. 42 d; P1, Flag-EV vs. Flag-TRIM22 vs. Raf-1^S338A^ vs. Flag-TRIM22 + Raf-1^S338A^: 47 d vs. 29.5 d vs. 43.5 d vs. 42 d; Fig. 6e). These results indicate that Raf-1 is a key effector by which TRIM22 promotes the growth of GBM cell populations in vitro and in vivo.

### TRIM22 promotes the K63-linked ubiquitination of ERK1/2

**Fig. 7:**
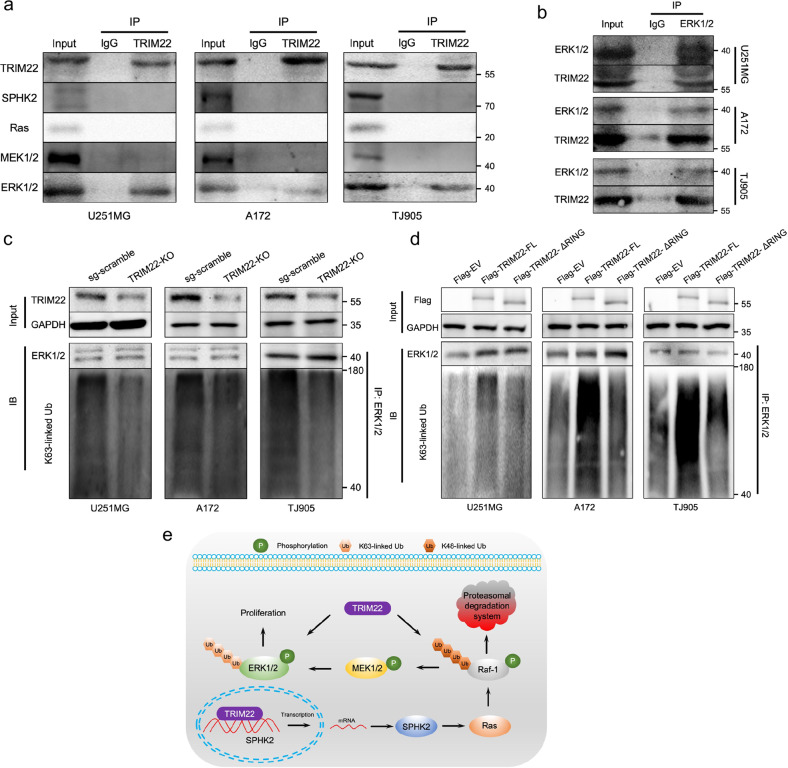
TRIM22 promotes the K63-linked ubiquitination of ERK1/2 through its RING domain. **a** Anti-TRIM22 antibody was used to detect the binding of TRIM22 to the SPHK2/MAPK pathway core protein in U251MG, A172 and TJ905 cells. **b** Association of TRIM22 with ERK1/2 in U251MG, A172 and TJ905 cells. **c, d** Western blot analysis for ERK1/2 of Co-IPs with K63-linkage specific polyubiquitin antibody in the indicated modified U251MG, A172, and TJ905 cells. **e** Graphical model for TRIM22-mediated SPHK2/MAPK activation and GBM growth. Each experiment was repeated three times.

*TRIM22* knockout decreased the expression of key proteins of the SPHK2/MAPK pathway, including SPHK2, Ras, phosphorylated MEK1/2, and ERK1/2 (Fig. 3b, Supplementary Fig. 5a), whereas overexpression of TRIM22 increased the expression of these proteins (Fig. 5b, Supplementary Fig. 7b). The parallel expression trends of these proteins and TRIM22 suggest that TRIM22 may directly regulate the activation of these proteins. To explore whether TRIM22 directly regulates these key proteins, an anti-TRIM22 antibody was used in the co-IP experiment. ERK1/2 was associated with TRIM22 in four GBM cell lines and two primary GBM cell lines (Fig. 7a, Supplementary Fig. 9a). The same results were obtained by co-IP with an anti-ERK1/2 antibody (Fig. 7b, Supplementary Fig. 9b). Furthermore, we found that K63-linked ubiquitination of ERK1/2 led to a parallel decrease ERK1/2 and TRIM22 protein levels. *TRIM22* knockout reduced the K63-linked ubiquitination levels of ERK1/2 (Fig. 7c, Supplementary Fig. 9c), and overexpression of TRIM22 increased the levels. This specific mechanism may also be related to the RING domain of TRIM22 (Fig. 7d, Supplementary Fig. 9d). These results suggest that TRIM22 may regulate MAPK activation through ERK1/2 activation.

### The RING domain and NLS of TRIM22 promote the proliferation of GBM

**Fig. 8:**
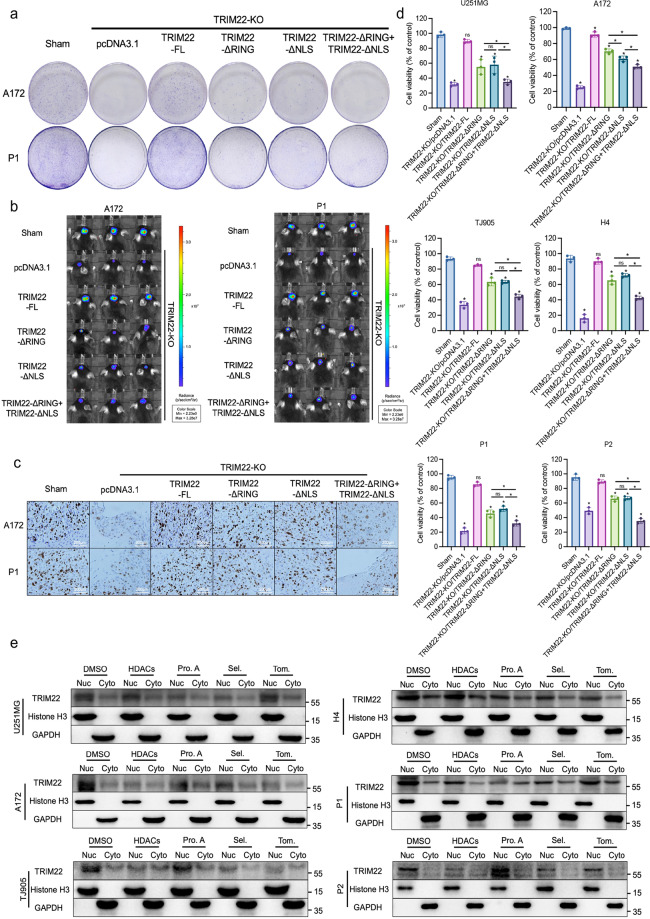
The RING domain and NLS are important regions involved in TRIM22-mediation promotion of GBM cell proliferation. **a** Detection of the colony formation ability of A172 and P1 cells by different modified TRIM22 mutants (*n* = 3 per group). **b** GBM cells modified by different TRIM22 mutants were injected into the brains of mice (*n* = 5 mice per group), and representative photos of fluorescence were taken one month after the operation. **c** Ki-67 index of tumor tissue one month after the operation in each group. **d** A CCK-8 assay was used to detect the activity difference of cells modified by different TRIM22 mutants (*n* = 3 per group). U251MG: F (5, 12) = 50.18, *P* < 0.0001, A172: F (5, 12) = 303.4, *P* < 0.0001, TJ905: F (5, 12) = 145.2, *P* < 0.0001, H4: F (5, 12) = 160,6, *P* < 0.0001, P1: F (5, 12) = 181.1, *P* < 0.0001, P2: F (5, 12) = 124.5, *P* < 0.0001. **e** WB was performed to examine the effect of inhibitors of different signaling pathways on TRIM22 nuclear translocation. 5 μM HDACis, 50 μM Pro. A, 10 nM Sel, and 30 μM Tom for 24 h. Pro. A: protosappanin A; Sel: selumetinib; Tom: tomatidine. The data were analyzed using one-way ANOVA, and all data are expressed as the mean ± standard deviation. *P < 0.05 represents a statistically significant difference between the two groups. ns, not significant. Each experiment was repeated three times.

The above experimental results revealed that TRIM22 acts as a transcription factor in the nucleus and regulates the modification of Raf-1 and ERK1/2 through its RING domain in the cytoplasm. To explore the effects of these mechanisms on GBM cell proliferation, TRIM22-ΔNLS and TRIM22-ΔRING plasmids were constructed. A172 and P1 cells were transfected with the different modified plasmids after TRIM22 was knocked out using CRISPR/Cas9 technology. TRIM22-KO significantly inhibited the formation of cell colonies. Transfection with the TRIM22-FL plasmid completely restored the proliferation inhibition induced by TRIM22-KO. Interestingly, transfection of the TRIM22 plasmid with the RING domain and NLS deletion did not completely restore the inhibition of proliferation caused by TRIM22-KO (Fig. 8a, Supplementary Fig. 10a). In the in situ tumorigenesis experiment in mice, the results for the fluorescence intensity (Fig. 8b, Supplementary Fig. 10b) and the Ki-67 index of the tumor body (Fig. 8c, Supplementary Fig. 10c) were consistent with the results of the colony formation experiment one month after tumorigenesis. We also assessed the effects of different modification groups on cell activity in vitro, and the results showed that the six cell lines of TRIM22-KO, TRIM22-ΔRING, and TRIM22-ΔNLS had weaker recovery effects on cell activity than the TRIM22-FL group (Fig. 8d). These experimental data indicate that the RING domain of TRIM22 and the transcription factor role of TRIM22 in the nucleus play a crucial role in the proliferation of GBM cells.

In addition, we explored whether nuclear localization was controlled by any extracellular signals using a variety of signaling pathway inhibitors, such as dual HDAC and mTOR inhibitors (HDACis), the JAK2/STAT3 inhibitor protosappanin A, the non-ATP-competitive MEK1/2 inhibitor selumetinib, and the NF-κB/JNK inhibitor tomatidine. The cell lines were differentially affected by the different signaling inhibitors. HDACis could not inhibit the nuclear translocation of TRIM22 in U251MG and H4 cells, while protosappanin A could not inhibit translocation in TJ905 and P2 cells. Only in U251MG cells did tomatidine not show an inhibitory effect. Interestingly, selumetinib significantly inhibited the nuclear translocation of TRIM22 in all detected cell lines, which was consistent with role of TRIM22 in the MEK1/2/ERK1/2 pathway revealed by our previous results (Fig. 8e, Supplementary Fig. 10d). These data indicate that TRIM22 nuclear localization is regulated by a variety of extracellular signaling pathways.

## Discussion

TRIM22 plays a key role as an E3 ubiquitin ligase in tumors. Our study revealed, for the first time, that TRIM22 regulates *SPHK2* transcription as a transcription factor in the nucleus. We found that the main binding sites of TRIM22 were located in intron 2 (the corresponding transcript is NM_001243876) and exon 2 (the corresponding transcript is NM_001204160). In addition, TRIM22 positively regulates the expression of SPHK2 and indirectly affects the activation of MAPK signaling. TRIM22 also directly binds to the MAPK pathway core proteins Raf-1 and ERK1/2, regulates the degradation of Raf-1 through K48-linked ubiquitination, and regulates the activation of ERK1/2 by K63-linked ubiquitination. In addition, deletion of the RING domain and nuclear localization sequence of TRIM22 significantly inhibited TRIM22-induced proliferation of GBM cells in vivo and in vitro. These results illustrate that TRIM22 directly or indirectly regulates MAPK signaling, thereby promoting GBM cell proliferation (Fig. 7e).

TRIM22 contains four domains: the RING, B-Box, CC, and SPRY domains. The RING domain is reported to be involved in ubiquitination as a classical domain of the TRIM family^15^. The specificity of the Ub binding system arises from the direct binding of E3 ligases to their substrates. Our finding that TRIM22 binds to Raf-1 and ERK1/2 and promotes K48-linked and K63-linked ubiquitination, respectively, is consistent with the functions reported in the literature. In addition, we found that the deletion of the RING domain significantly inhibited SPHK2/MAPK signaling activation by causing overexpression of TRIM22, suggesting that the RING domain of TRIM22 may regulate signaling activation in addition to acting as an E3 ubiquitin ligase. Although the RING domain plays a functionally important role and has been shown to be the main domain promoting proliferation in GBM, the binding of TRIM22 to Raf-1 does not appear to be related to the RING domain. Studies have revealed that TRIM22 binds IκBα by association with its B-Box, CC, and SPRY domains^21^, while our study revealed that the binding of TRIM22 and Raf-1 is associated with the CC and SPRY domains. On the other hand, we found that TRIM22 functions independently of these four domains when it acts as a transcription factor to regulate *SPHK2* transcription. Polymorphisms affecting the function of TRIM22; thus, it is necessary to further investigate which region of TRIM22 may be associated with the development of human gliomas.

Raf-1 is a major effector recruited through the GTP-binding protein Ras that activates the MEK-MAPK pathway^23^. Activation of Raf-1 involves the phosphorylation of multiple activation sites, including Ser338, Tyr341, Thr491, Ser494, Ser497, and Ser499^22^. The ratio of Raf-1^S338^/Raf-1 illustrates the activation of Raf-1, and our study revealed that the synergistic effect of TRIM22 on both Raf-1 phosphorylation and degradation prompted Raf activation. In addition, the p44/42 MAPK (ERK1/2) signaling pathway is activated in response to various extracellular stimuli. MEK1 and MEK2 activate p44 and p42, respectively, by phosphorylating the activation loop residues Thr202/Tyr204 and Thr185/Tyr187, respectively^24,25^. ERK1/2 is a key target for cancer diagnosis and treatment^26^. We demonstrated that TRIM22 targets Raf-1 for proteasome-mediated degradation and further enhances MAPK signaling activation by modifying the K63-linked polyubiquitin chains on ERK1/2. However, in addition to K48- and K63-linked chains, K6-, K11-, K27-, K29-, and K33-linked polyubiquitin chains also play important roles in the regulation of Raf-1 and ERK1/2; however, whether TRIM22 regulates other ubiquitination and phosphorylation sites of Raf-1 and ERK1/2 also needs further study.

K145 is a selective inhibitor of SPHK2 and shows excellent antitumor activity in vivo and in vitro^27,28^. Liu’s group^29^ found that K145 can act as a dual pathway inhibitor and inhibit both ART and AKT signaling pathway phosphorylation while inhibiting SPHK2, thereby achieving antiproliferative and apoptotic effects in U937 leukemia cells. Selumetinib is a highly potent and selective non-ATP-competitive MEK1/2 inhibitor^30^, and the results from the National Cancer Institute showed a good therapeutic effect^31^ in a clinical trial of selumetinib in the treatment of low-grade astroglioma and metastatic low-grade glioma (NCT04166409) conducted on January 3, 2020. Our study revealed that K145 and selumetinib significantly inhibited TRIM22 overexpression-induced proliferation of GBM cells both in vivo and in vitro.

In addition, we found that TRIM22 was localized in the nucleus and therefore hypothesized that TRIM22 may function as a transcription factor to regulate the transcription of genes. The ChIP-Seq results showed that TRIM22 may regulate the transcription of SPHK2, but peak is not located in the gene promoter region. SPHK2 (NM_001204160) has 6 exons and 5 introns in total. The peak covers most of exon 2 and some of intron 2. Typically, transcription factors bind to promoter regions of genes to regulate gene transcription. However, it has been reported that modifications of histones in the gene body region are associated with transcriptional activation of genes^32–34^. Genomic DNA is not completely encapsulated by histones, so some DNA is exposed. We speculate that TRIM22 may bind to exposed *SPHK2* DNA while regulating nearby histones, thus affecting the transcription of SPHK2. This mechanism could also explain the negative results obtained for SPHK2 regulation by different TRIM22 truncates in yeast one-hybrid experiments. However, specific and detailed regulatory patterns remain to be further investigated.

Our study revealed that both the RING structure and transcription factor activity of TRIM22 affected GBM cell proliferation. We generated TRIM22 mutant constructs lacking the NLS sequence to inhibit the nuclear translocation of TRIM22. The NLS is located in the 257-275 aa region of TRIM22, which does not overlap with the four domains of TRIM22. Therefore, although both the NLS and RING domains promote TRIM22-induced GBM cell proliferation, the deletion of either one of the two regions inhibited the proliferation of GBM cells.

In addition, we could not identify the mouse homolog of *TRIM22* in NCBI, and unfortunately, we cannot use a spontaneous tumor mouse model (for example, Pten, Nf1, and p53 are conditionally knocked out in astrocytes). TRIM22 has been shown to be a p53 target gene. As a tumor suppressor gene, p53 is closely associated with tumorigenesis and proliferation. Our research focused on the role of TRIM22 as an E3 ubiquitin enzyme and transcription factor, which is not directly related to p53. Normal A172 cells are not tumorigenic in vivo. Therefore, the malignant GBM cells used in our xenograft model (A172 and P1) expressed mutant p53^35,36^. This also excludes the possible interference of p53 in our experimental results.

Our results showed that TRIM22 may play a role in promoting GBM cell proliferation through the SPHK2/MAPK signaling pathway. The nuclear translocation of TRIM22 is related to the proliferation of GBM cells, but many other signaling pathway (mTOR, JAK2/STAT3, NF-κB/JNK) inhibitors can also affect the nuclear translocation of TRIM22 to some extent. As shown in Fig. 8e, different signaling pathway inhibitors have distinct effects on different cells. These results indicate that TRIM22 may have a more complex regulatory mechanism in glioma and highlight the importance of personalized treatment for cancer patients. In addition, heterogeneity in gliomas also depends on molecular characteristics, such as MGMT status, IDH, and ARTX. We found that the expression of TRIM22 was positively correlated with glioma grade, while there were multiple differences in the molecular profiles in different grades of glioma. Therefore, it is necessary to take into account the association between these molecular features and TRIM22 in future work to further investigate the function and mechanism of TRIM22 in gliomas.

In conclusion, our study revealed for the first time an entirely new regulatory mechanism of MAPK activation, revealing that TRIM22, which is highly expressed in GBM, regulates the activation of MAPK signaling by both direct (E3 ubiquitin ligase) and indirect (transcription factor) means. TRIM22 induces SPHK2/MAPK signaling activation in GBM, driving tumor growth and progression. Finally, our study identified TRIM22 as a candidate therapeutic target. Drug inhibition of E3 ligase activity or the TRIM22/SPHK2/MAPK interaction may provide a promising strategy for treating GBM.

## Supplementary information

supplementary information

## Data Availability

The datasets used and/or analyzed during the current study are available from the corresponding author on reasonable request.

## References

[1] Baccarini M (2005). Second nature: biological functions of the Raf-1 “kinase”. FEBS Lett..

[2] Meloche S, Pouysségur J (2007). The ERK1|[sol]|2 mitogen-activated protein kinase pathway as a master regulator of the G1- to S-phase transition. Oncogene.

[3] Roux PP, Blenis J (2004). ERK and p38 MAPK-activated protein kinases: a family of protein kinases with diverse biological functions. Microbiol. Mol. Biol. Rev..

[4] Daniel P, Filiz G, Mantamadiotis T (2016). Sensitivity of GBM cells to cAMP agonist-mediated apoptosis correlates with CD44 expression and agonist resistance with MAPK signaling. Cell Death Dis..

[5] Chen X (2020). Activation of JNK and p38 MAPK mediated by ZDHHC17 drives glioblastoma multiforme development and malignant progression. Theranostics.

[6] Xu Y (2020). RND2 attenuates apoptosis and autophagy in glioblastoma cells by targeting the p38 MAPK signalling pathway. J. Exp. Clin. Cancer Res..

[7] Vitucci M (2013). Cooperativity between MAPK and PI3K signaling activation is required for glioblastoma pathogenesis. Neuro. Oncol..

[8] Shi D, Grossman S (2010). Ubiquitin becomes ubiquitous in cancer: emerging roles of ubiquitin ligases and deubiquitinases in tumorigenesis and as therapeutic targets. Cancer Biol. Ther..

[9] Hatakeyama S (2011). TRIM proteins and cancer. Nat. Rev. Cancer.

[10] Akutsu M, Dikic I, Bremm A (2016). Ubiquitin chain diversity at a glance. J. Cell Sci..

[11] Swatek KN, Komander D (2016). Ubiquitin modifications. Cell Res..

[12] Bigenzahn J (2018). LZTR1 is a regulator of RAS ubiquitination and signaling. Science.

[13] Sato T, Takahashi H, Hatakeyama S, Iguchi A, Ariga T (2015). The TRIM-FLMN protein TRIM45 directly interacts with RACK1 and negatively regulates PKC-mediated signaling pathway. Oncogene.

[14] Yin H (2017). GPER promotes tamoxifen-resistance in ER+ breast cancer cells by reduced Bim proteins through MAPK/Erk-TRIM2 signaling axis. Int. J. Oncol..

[15] Duan Z, Gao B, Wei X, Xiong S (2008). Identification of TRIM22 as a RING finger E3 ubiquitin ligase. Biochem. Biophys. Res. Commun..

[16] Bo G, Duan Z, Wei X, Xiong S (2010). Tripartite motif-containing 22 inhibits the activity of hepatitis B virus core promoter, which is dependent on nuclear-located RING domain. Hepatology.

[17] Zhang F, Hu W, Qu L, Cang C (2020). Sphingosine kinase 2 inhibitor ABC294640 suppresses neuronal excitability and inhibits multiple endogenously and exogenously expressed voltage-gated ion channels in cultured cells. Channels (Austin).

[18] Dymond A (2017). Effects of cytochrome P450 (CYP3A4 and CYP2C19) inhibition and induction on the exposure of selumetinib, a MEK1/2 inhibitor, in healthy subjects: results from two clinical trials. Eur. J. Clin. Pharmacol..

[19] Fei X (2019). Eupatilin inhibits glioma proliferation, migration, and invasion by arresting cell cycle at G1/S phase and disrupting the cytoskeletal structure. Cancer Manag. Res..

[20] Fei X (2019). The role of Toll-like receptor 4 in apoptosis of brain tissue after induction of intracerebral hemorrhage. J. Neuroinflammation.

[21] Ji J (2021). TRIM22 activates NF-κB signaling in glioblastoma by accelerating the degradation of IκBα. Cell Death Differ..

[22] Chong H, Lee J, Guan K (2001). Positive and negative regulation of Raf kinase activity and function by phosphorylation. EMBO J..

[23] Avruch J, Zhang X, Kyriakis J (1994). Raf meets Ras: completing the framework of a signal transduction pathway. Trends Biochem. Sci..

[24] Rubinfeld H, Seger R (2005). The ERK cascade: a prototype of MAPK signaling. Mol. Biotechnol..

[25] Murphy LO, Blenis J (2006). MAPK signal specificity: the right place at the right time. Trends Biochem. Sci..

[26] Roberts P, Der C (2007). Targeting the Raf-MEK-ERK mitogen-activated protein kinase cascade for the treatment of cancer. Oncogene.

[27] LeBlanc F (2020). Sphingosine kinase-2 is overexpressed in large granular lymphocyte leukemia and promotes survival through Mcl-1. Br. J. Haematol..

[28] Zhang R, Li L, Yuan L, Zhao M (2016). Hypoxic preconditioning protects cardiomyocytes against hypoxia/reoxygenation-induced cell apoptosis via sphingosine kinase 2 and FAK/AKT pathway. Exp. Mol. Pathol..

[29] Liu, K. et al. Biological characterization of 3-(2-amino-ethyl)-5-[3-(4-butoxyl-phenyl)-propylidene]-thiazolidine-2,4-dione (K145) as a selective sphingosine kinase-2 inhibitor and anticancer agent. *PLoS ONE* **8**, e56471 (2013). 10.1371/journal.pone.0056471 PMC3577900 23437140

[30] Dymond A (2017). Pharmacokinetics and pharmacogenetics of the MEK1/2 inhibitor, selumetinib, in Asian and Western healthy subjects: a pooled analysis. Eur. J. Clin. Pharmacol..

[31] MEK1/2 inhibition is effective in subsets of pediatric low-grade glioma. *Cancer Discov*. **9**, OF11 (2019). http://cancerdiscovery.aacrjournals.org/CDNews.

[32] Zhu Q (2011). BRCA1 tumour suppression occurs via heterochromatin-mediated silencing. Nature.

[33] Minsky N (2008). Monoubiquitinated H2B is associated with the transcribed region of highly expressed genes in human cells. Nat. Cell Biol..

[34] Shema E (2008). The histone H2B-specific ubiquitin ligase RNF20/hBRE1 acts as a putative tumor suppressor through selective regulation of gene expression. Genes Dev..

[35] Badie B, Goh CS, Klaver J, Herweijer H, Boothman DA (1999). Combined radiation and p53 gene therapy of malignant glioma cells. Cancer Gene Ther..

[36] Uzzaman M, Keller G, Germano IM (2009). In vivo gene delivery by embryonic-stem-cell-derived astrocytes for malignant gliomas. Neuro. Oncol..

