# Supplementary material for: TRIM22 promotes the proliferation of glioblastoma cells by activating MAPK signaling and accelerating the degradation of Raf-1
Source: Exp Mol Med. 2023 Jun 1;55(6):1203–17. doi: 10.1038/s12276-023-01007-y (PMC10318069; doi:10.1038/s12276-023-01007-y)
Supplement: Supplementary file 1 — supplementary information [file 12276_2023_1007_MOESM1_ESM.pdf]

Experimental and Molecular Medicine

Supplementary Materials for

TRIM22 promotes the proliferation of glioblastoma cells by activating MAPK signaling and accelerating the degradation of Raf-1

Xiaowei Fei[1*], Ya-nan Dou[1*], Kai Sun[2*], Jialiang Wei[1], Qingdong Guo[1], Li Wang[1], Xiuquan Wu[1], Weihao Lv[1], Xiaofan Jiang[1#], Zhou Fei[1#]

[1]Department of Neurosurgery, Xijing Hospital, Air Force Military Medical University, Xi'an, Shaanxi, 710032, China.

[2]Department of Neurosurgery, Daping Hospital, Third Military Medical University, Chongqing 400042, China.

*These authors contributed equally to this work.

[#]Correspondence should be addressed to Xiaofan Jiang (jiangxiaofan123123@163.com) and Zhou Fei (feizhou123123@163.com)

Xijing Hospital, Air Force Military Medical University

No. 127, Changle West Road, Xincheng District, Shaanxi, 710032, China.

Tel: +86-18829285387

Fax: Not application.

This PDF file includes:

Materials and Methods

Supplementary Fig. 1 to 10

Supplementary Table 1 to 4

Materials and Methods

Chromatin immunoprecipitation sequencing and qPCR (ChIP-seq and ChIP-qPCR)

10 ng of DNA samples were prepared for Illumina sequencing as the following steps: 1) DNA samples were blunt-ended; 2) AdA base was added to the 3' end of each strand; 3) Illumina's genomic adapters were ligated to the DNA fragments; 4) PCR amplification was performed to enrich ligated fragments; 5) Size selection of ~200-1500bp enriched product using AMPure XP beads. The completed libraries were quantified by Agilent 2100 Bioanalyzer. The libraries were denatured with 0.1 M NaOH to generate single-stranded DNA molecules, captured on Illumina flow cell, amplified in situ. The libraries were then sequenced on the Illumina NovaSeq 6000 following the NovaSeq 6000 S4 Reagent Kit (300 cycles) protocol. After the sequencing platform generated the sequencing images, the stages of image analysis and base calling were performed using Off-Line Basecaller software (OLB V1.8). Sequence quality was examined using the FastQC software. After passing Solexa CHASTITY quality filter, the clean reads were aligned to Human genome (UCSC HG19) using BOWTIE software (V2.1.0). Aligned reads were used for peak calling of the ChIP regions using MACS V1.4.2. Statistically significant ChIP-enriched regions (peaks) were identified by comparison of IP vs Input or comparison to a Poisson background model, using a p-value threshold of 10^{-4}. The peaks in samples were annotated by the nearest gene using the newest UCSC RefSeq database.

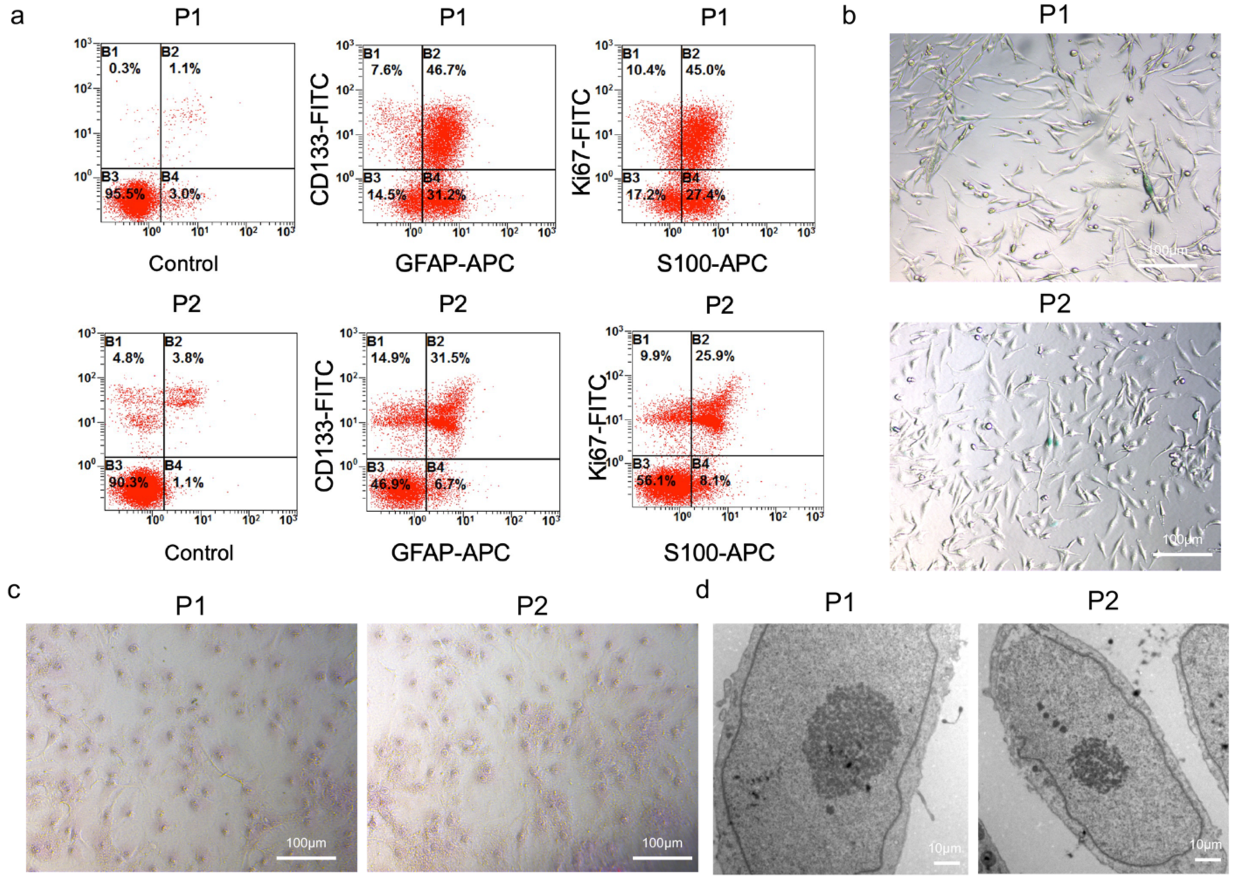

Supplementary Fig. 1 Identification of primary GBM cells P1 and P2.

a Flow cytometry was used to detect expression of GBM markers GFAP, S100, Ki67, and CD133 in P1 and P2 cells. **b** Detection of senescence in P1 and P2 cells. **c** HE staining of P1 and P2 cells. **d** Scanning electron microscopy was performed to detect subcellular structures of P1 and P2 cells.

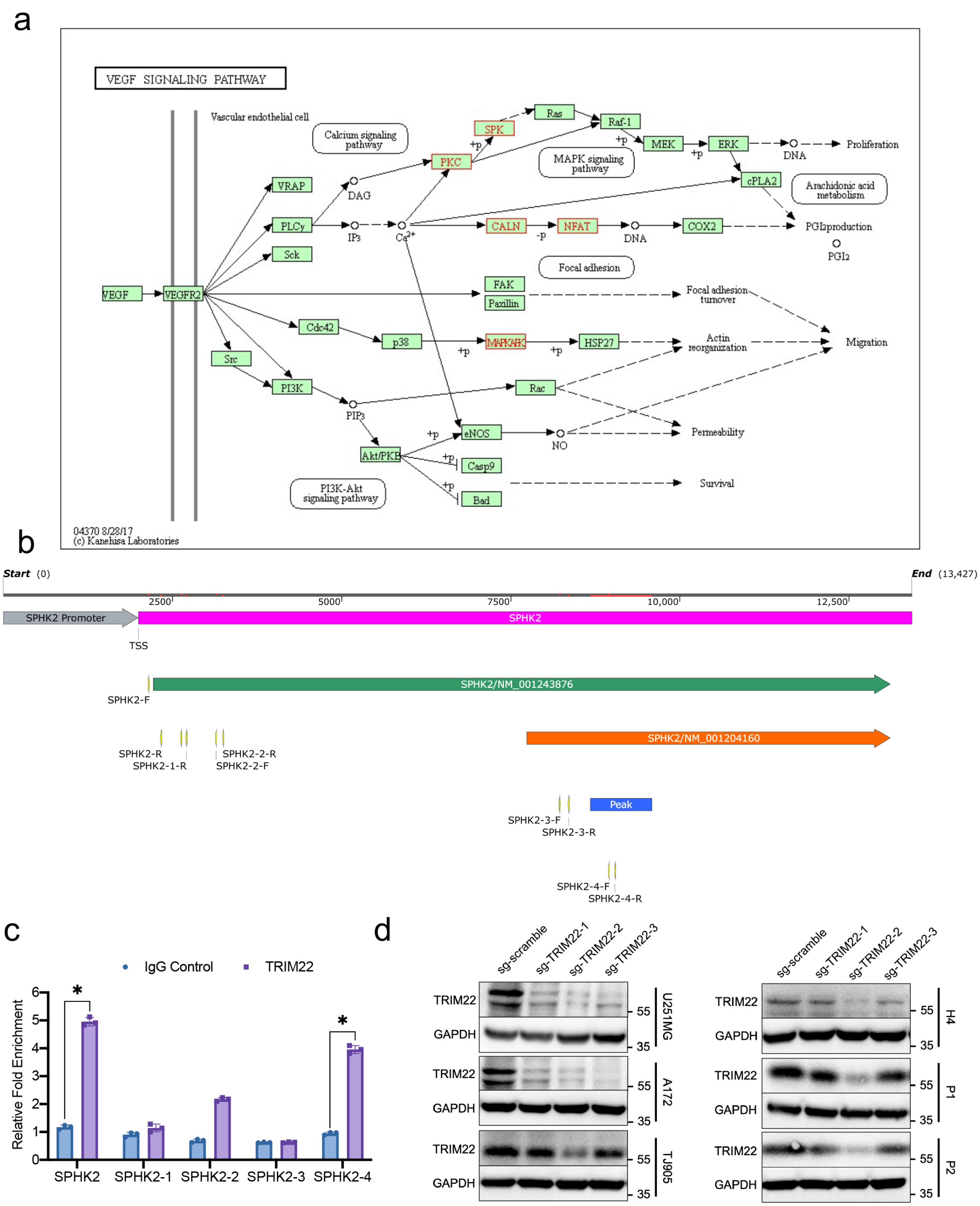
a
VEGF SIGNALING PATHWAY
Vascular endothelial cell
Calcium signaling pathway
MAPK signaling pathway
Arachidonic acid metabolism
PI3K-Akt signaling pathway
VEGF
VEGFR2
VRAP
PLCγ
Sck
Src
Cdc42
PI3K
p38
SPK
PKC
Ras
Raf-1
MEK
ERK
cPLA2
CALN
NFAT
COX2
FAK
Paxillin
MAPKAPK
HSP27
Rac
eNOS
Akt/PKB
Casp9
Bad
DAG
IP3
Ca2+
PIP3
NO
DNA
Proliferation
PGI2production
PGI2
Focal adhesion
Focal adhesion turnover
Actin reorganization
Migration
Permeability
Survival
04370 8/28/17
(c) Kanehisa Laboratories
b
Start (0)
End (13,427)
2500
5000
7500
10,000
12,500
SPHK2 Promoter
SPHK2
TSS
SPHK2/NM_001243876
SPHK2-F
SPHK2/NM_001204160
SPHK2-R
SPHK2-1-R
SPHK2-2-R
SPHK2-2-F
Peak
SPHK2-3-F
SPHK2-3-R
SPHK2-4-F
SPHK2-4-R
c
IgG Control
TRIM22
Relative Fold Enrichment
SPHK2
SPHK2-1
SPHK2-2
SPHK2-3
SPHK2-4
d
sg-scramble
sg-TRIM22-1
sg-TRIM22-2
sg-TRIM22-3
TRIM22
GAPDH
U251MG
A172
TJ905
H4
P1
P2
55
35

Supplementary Fig. 2 TRIM22 regulates SPHK2 transcription and affects MAPK signaling pathway.

a VEGF signaling pathway in KEGG analysis. **b** According to the ChIP-Seq results, SPHK2 primers were designed in segments. Pink box represents SPHK2 gene and gray box is the 2000 BP promoter region upstream of SPHK2. Green box represents a transcript of SPHK2 NM_001243876, orange box represents another transcript NM_001204160, blue box represents the position of peak in ChIP-Seq results. The above gene sequence and transcript sequence information refer to GRCh37/hg19 genome version information, and the sequence is obtained from UCSC database (UCSC Genome Browser, http://genome.ucsc.edu/index.html). NM_001243876 has 6 exons and 5 introns in total, and peak is located in its intron 2 region. NM_001204160 has 6 exons and 5 introns in total. Peak is located in most of the sequences of exon 2 and part of the sequences of intron 2. According to the Peak site, NM_001204160 was selected as the study subject. Detailed site information is shown in Supplementary Information: Table 3. **c** ChIP-qPCR detection of pull-down DNA (n = 3 per group). $F_{interaction}(4, 20) = 487.0$, $P<0.0001$. **d** Verification of *TRIM22* knockout efficiency. The data were analyzed using one-way and all data are expressed as the mean ± standard deviation. *$P < 0.05$ represents a statistically significant difference between the two groups. Each experiment was repeated three times.

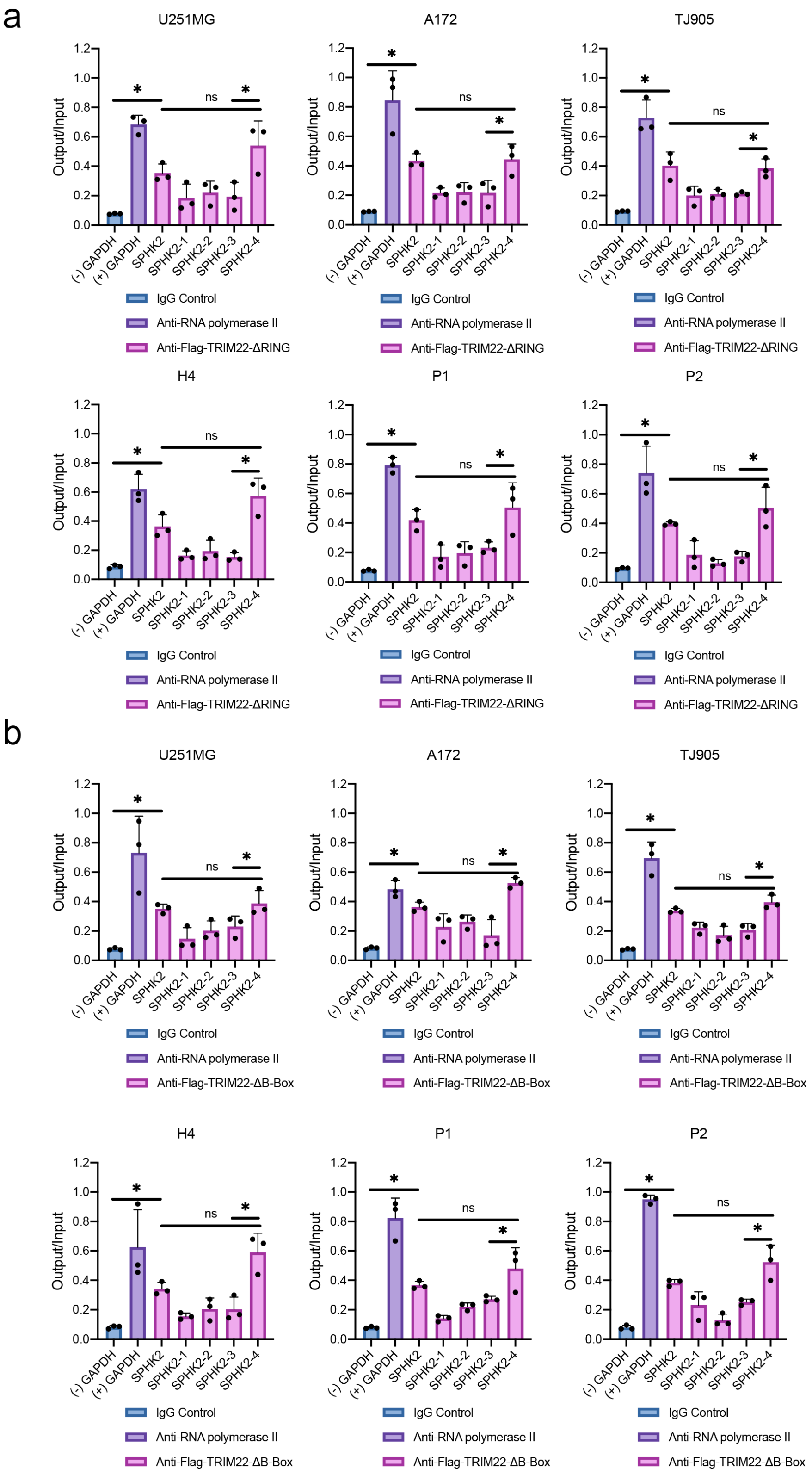

a
U251MG
A172
TJ905
H4
P1
P2
Output/Input
0.0
0.2
0.4
0.6
0.8
1.0
1.2
*
ns
(-) GAPDH
(+) GAPDH
SPHK2
SPHK2-1
SPHK2-2
SPHK2-3
SPHK2-4
IgG Control
Anti-RNA polymerase II
Anti-Flag-TRIM22-ΔRING
b
U251MG
A172
TJ905
H4
P1
P2
Output/Input
*
ns
(-) GAPDH
(+) GAPDH
SPHK2
SPHK2-1
SPHK2-2
SPHK2-3
SPHK2-4
IgG Control
Anti-RNA polymerase II
Anti-Flag-TRIM22-ΔB-Box

Supplementary Fig. 3 TRIM22 binds to the transcriptional region of *SPHK2*.

Different binding sites of truncated TRIM22 and *SPHK2 (1-4)* were detected by CUT&Tag in U251MG, A172, TJ905, H4 and primary GBM cells P1 and P2 transfected with Flag-TRIM22-ΔRING (**a**) (n = 3 per group) or Flag-TRIM22-ΔB-Box (**b**) (n = 3 per group). (**a**) U251MG: $F (6, 14) = 16.56$, $P<0.0001$; A172: $F (6, 14) = 20.30$, $P<0.0001$; TJ905: $F (6, 14) = 28.90$, $P<0.0001$; H4: $F (6, 14) = 24.93$, $P<0.0001$; P1: $F (6, 14) = 25.92$, $P<0.0001$; P2: $F (6, 14) = 18.77$, $P<0.0001$. (**b**) U251MG: $F (6, 14) = 11.48$, $P=0.0001$; A172: $F (6, 14) = 20.36$, $P<0.0001$; TJ905: $F (6, 14) = 40.79$, $P<0.0001$; H4: $F (6, 14) = 9.946$, $P=0.0002$; P1: $F (6, 14) = 32.32$, $P<0.0001$; P2: $F (6, 14) = 73.44$, $P<0.0001$. The data were analyzed using one-way analysis of variance and all data are expressed as the mean ± standard deviation. *$P < 0.05$ represents a statistically significant difference between the two groups. ns, not significant. Each experiment was repeated three times.

a

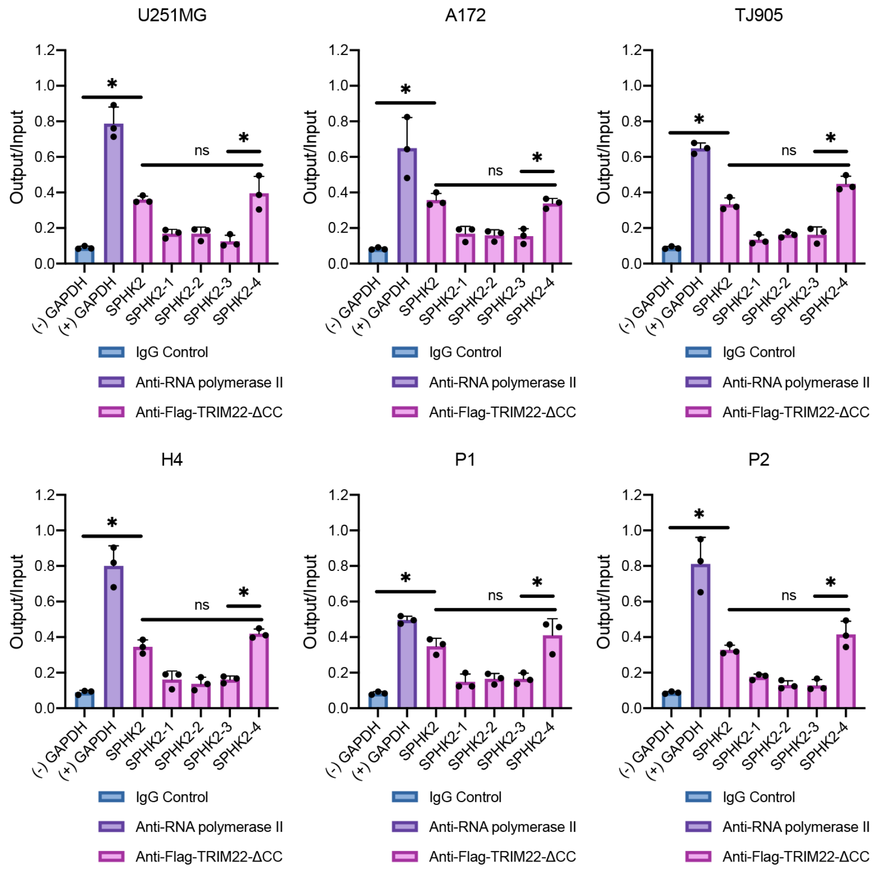
U251MG
A172
TJ905
H4
P1
P2
Output/Input
1.2
1.0
0.8
0.6
0.4
0.2
0.0
(-) GAPDH
(+) GAPDH
SPHK2
SPHK2-1
SPHK2-2
SPHK2-3
SPHK2-4
ns
*
IgG Control
Anti-RNA polymerase II
Anti-Flag-TRIM22-ΔCC

b

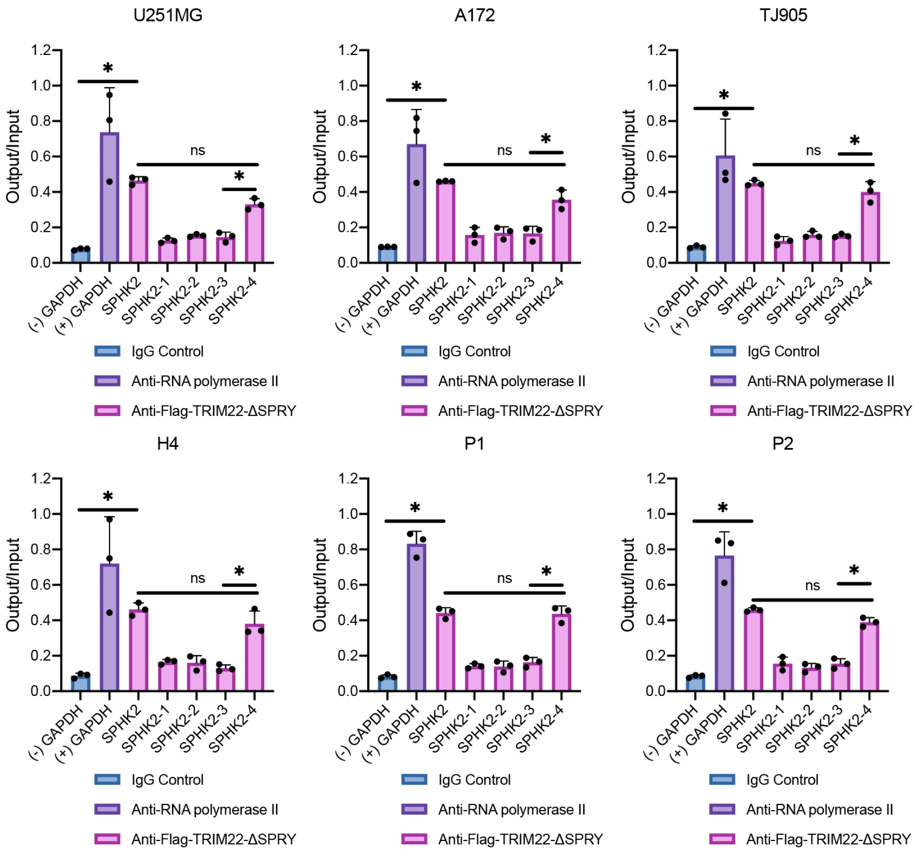
U251MG
A172
TJ905
H4
P1
P2
Output/Input
1.2
1.0
0.8
0.6
0.4
0.2
0.0
(-) GAPDH
(+) GAPDH
SPHK2
SPHK2-1
SPHK2-2
SPHK2-3
SPHK2-4
ns
*
IgG Control
Anti-RNA polymerase II
Anti-Flag-TRIM22-ΔSPRY

Supplementary Fig. 4 TRIM22 binds to the transcriptional region of *SPHK2*.

Different binding sites of truncated TRIM22 and *SPHK2 (1-4)* were detected by CUT&Tag in U251MG, A172, TJ905, H4 and primary GBM cells P1 and P2 transfected with Flag-TRIM22-ΔCC (**a**) (n = 3 per group) or Flag-TRIM22-ΔSPRY (**b**) (n = 3 per group). (**a**) U251MG: $F (6, 14) = 59.21$, $P<0.0001$; A172: $F (6, 14) = 21.90$, $P<0.0001$; TJ905: $F (6, 14) = 126.3$, $P<0.0001$; H4: $F (6, 14) = 68.87$, $P<0.0001$; P1: $F (6, 14) = 34.30$, $P<0.0001$; P2: $F (6, 14) = 44.84$, $P<0.0001$. (**b**) U251MG: $F (6, 14) = 18.17$, $P=0.0001$; A172: $F (6, 14) = 20.51$, $P<0.0001$; TJ905: $F (6, 14) = 18.07$, $P<0.0001$; H4: $F (6, 14) = 14.25$, $P<0.0001$; P1: $F (6, 14) = 155.1$, $P<0.0001$; P2: $F (6, 14) = 59.27$, $P<0.0001$. The data were analyzed using one-way analysis of variance and all data are expressed as the mean ± standard deviation. $^*P < 0.05$ represents a statistically significant difference between the two groups. ns, not significant. Each experiment was repeated three times.

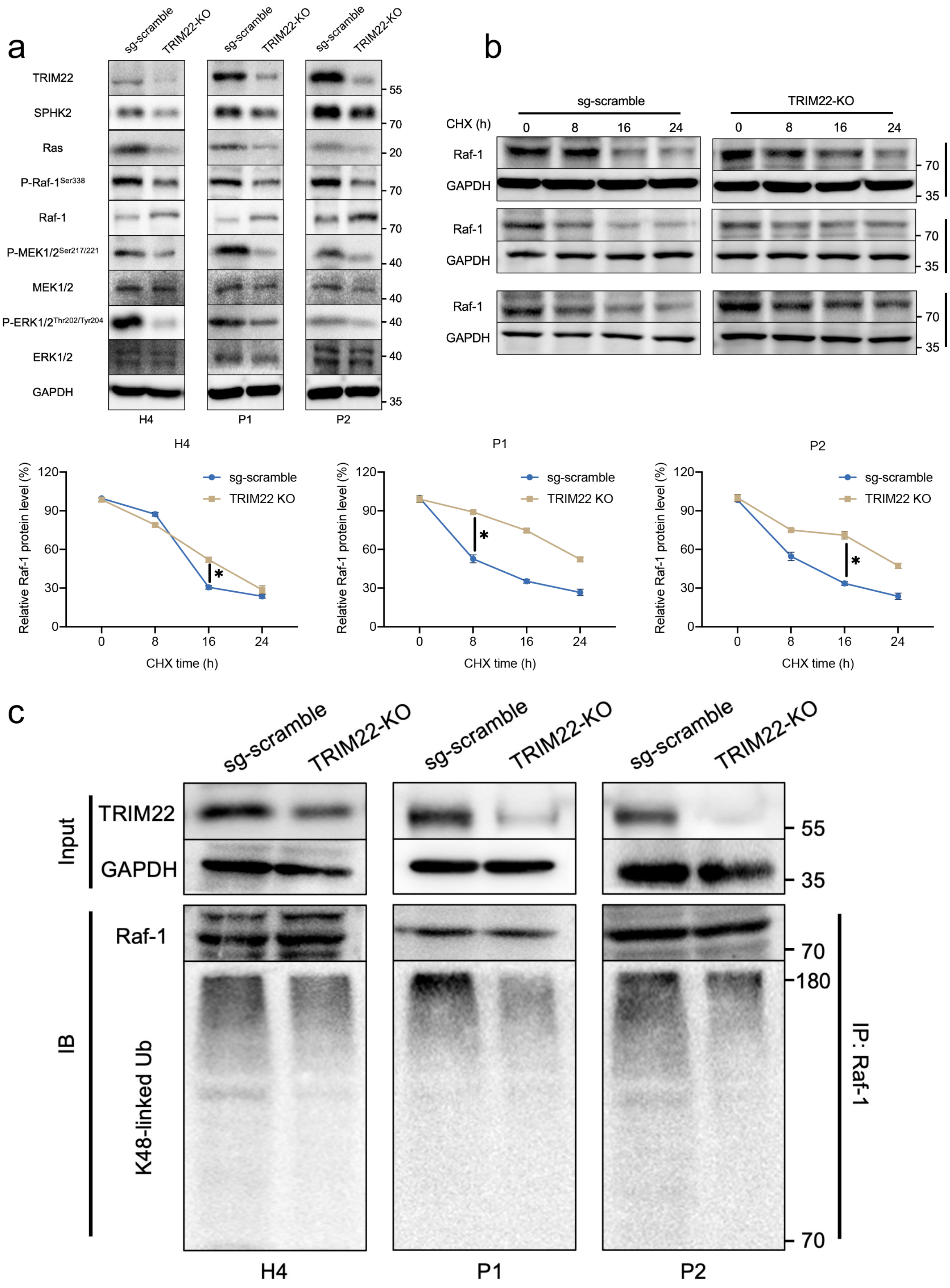
a
sg-scramble
TRIM22-KO
TRIM22
SPHK2
Ras
P-Raf-1Ser338
Raf-1
P-MEK1/2Ser217/221
MEK1/2
P-ERK1/2Thr202/Tyr204
ERK1/2
GAPDH
H4
P1
P2
b
sg-scramble
TRIM22-KO
CHX (h)
0
8
16
24
Raf-1
GAPDH
H4
P1
P2
Relative Raf-1 protein level (%)
CHX time (h)
sg-scramble
TRIM22 KO
c
Input
IB
TRIM22
GAPDH
Raf-1
K48-linked Ub
IP: Raf-1
H4
P1
P2

Supplementary Fig. 5 ***TRIM22*** **delete inhibit SPHK2/MAPK signaling via K48-linked ubiquitination of Raf-1.**

a The expression of SPHK2/MAPK pathway core protein was detected by Western blot. **b** Western blot analysis and quantification of Raf-1 protein in *TRIM22-KO* cells treated with cycloheximide (CHX; 25 μg/mL) for 0, 8, 16, and 24 h (n = 3 per group). H4: $F_{interaction}(3, 16) = 68.33$, $P<0.0001$; P1: $F_{interaction}(3, 16) = 127.6$, $P<0.0001$; P2: $F_{interaction}(3, 16) = 60.74$, $P<0.0001$. **c** IP experiment was used to detect the endogenous K48-linked ubiquitination of Raf-1 after *TRIM22* knockout. The data were analyzed using two-way analysis of variance and all data are expressed as the mean ± standard deviation. $^{*}P < 0.05$ represents a statistically significant difference between the two groups. Each experiment was repeated three times.

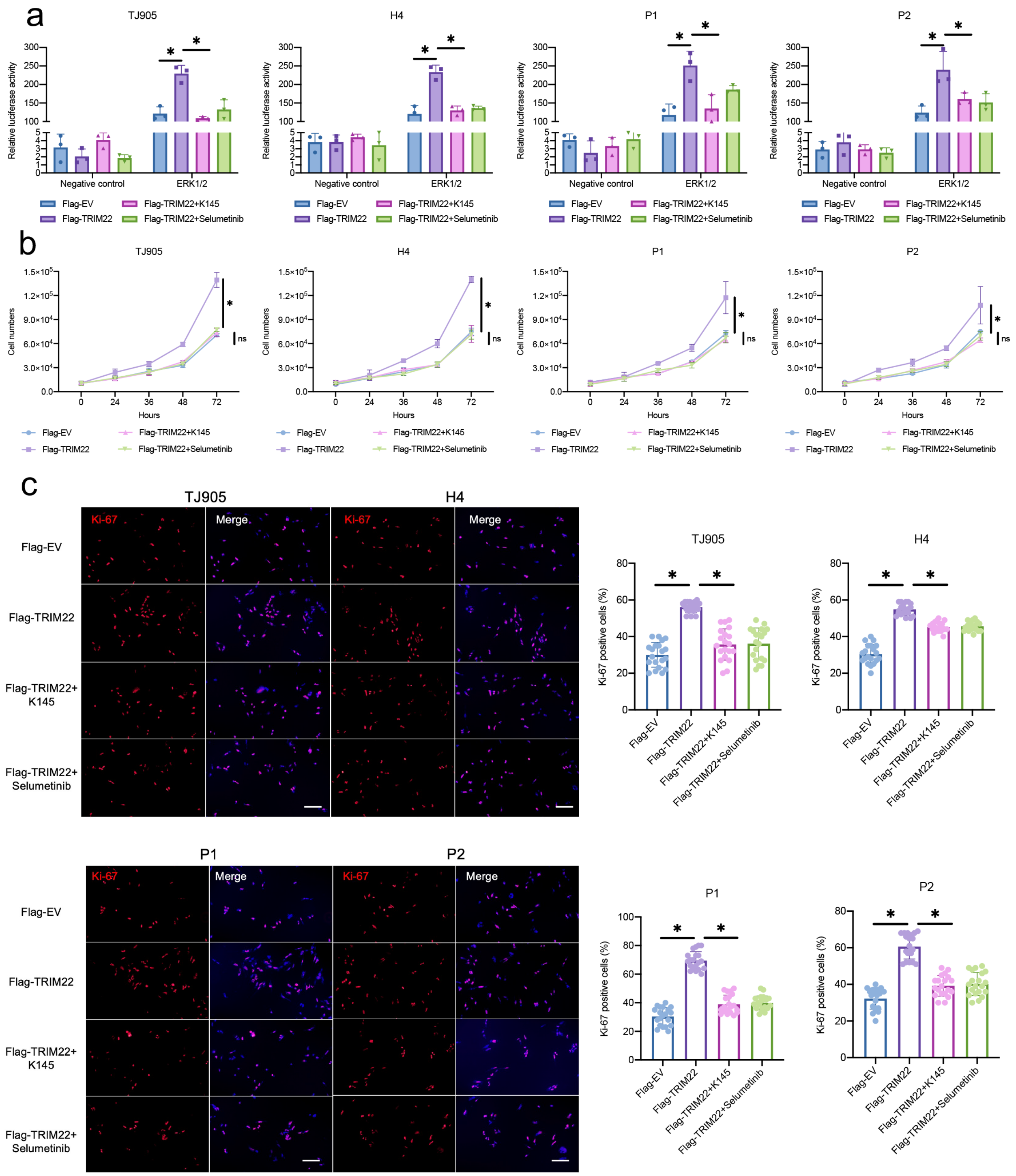
a
TJ905
H4
P1
P2
Relative luciferase activity
Negative control
ERK1/2
Flag-EV
Flag-TRIM22
Flag-TRIM22+K145
Flag-TRIM22+Selumetinib
b
Cell numbers
Hours
ns
c
Ki-67
Merge
Flag-EV
Flag-TRIM22
Flag-TRIM22+ K145
Flag-TRIM22+ Selumetinib
Ki-67 positive cells (%)

Supplementary Fig. 6 SPHK2/MAPK pathway regulates the proliferation of GBM in vivo and in vitro.

a Luciferase activity from TJ905[$F_{interaction}(3, 16) = 24.69$, P<0.0001], H4[$F_{interaction}(3, 16) = 31.99$, P<0.0001], P1[$F_{interaction}(3, 16) = 11.44$, P=0.0003] and P2[$F_{interaction}(3, 16) = 8.177$, P=0.0016] treated with K145 and Selumetinib, along with a reporter plasmid carrying the ERK1/2 promoter relative to negative control (n = 3 per group). **b** Growth curves generated using cell counting over 72 h for the cells indicated (n = 3 per group). TJ905: $F_{interaction}(12, 40) = 43.64$, P<0.0001; H4: $F_{interaction}(12, 40) = 33.51$, P<0.0001; P1: $F_{interaction}(12, 40) = 10.36$, P<0.0001; P2: $F_{interaction}(12, 40) = 5.415$, P<0.0001. **c** Representative images and quantification of Ki-67 immunofluorescence staining from modified TJ905 [F (3, 76) = 52.08, P<0.0001], H4 [F (3, 76) = 159.9, P<0.0001], P1 [F (3, 76) = 161.8, P<0.0001] and P2 [F (3, 76) = 80.22, P<0.0001] cells (n = 20 per group). Scale bar: 50 μm. In vitro experiment, Cells were treated with 5 μM K145 or 10 nM selumetinib for 24 hours. The data were analyzed using one-way ANOVA (**c**) or two-way ANOVA (**a** and **b**) and all data are expressed as the mean ± standard deviation. *P < 0.05 represents a statistically significant difference between the two groups. ns, not significant. Each experiment was repeated three times.

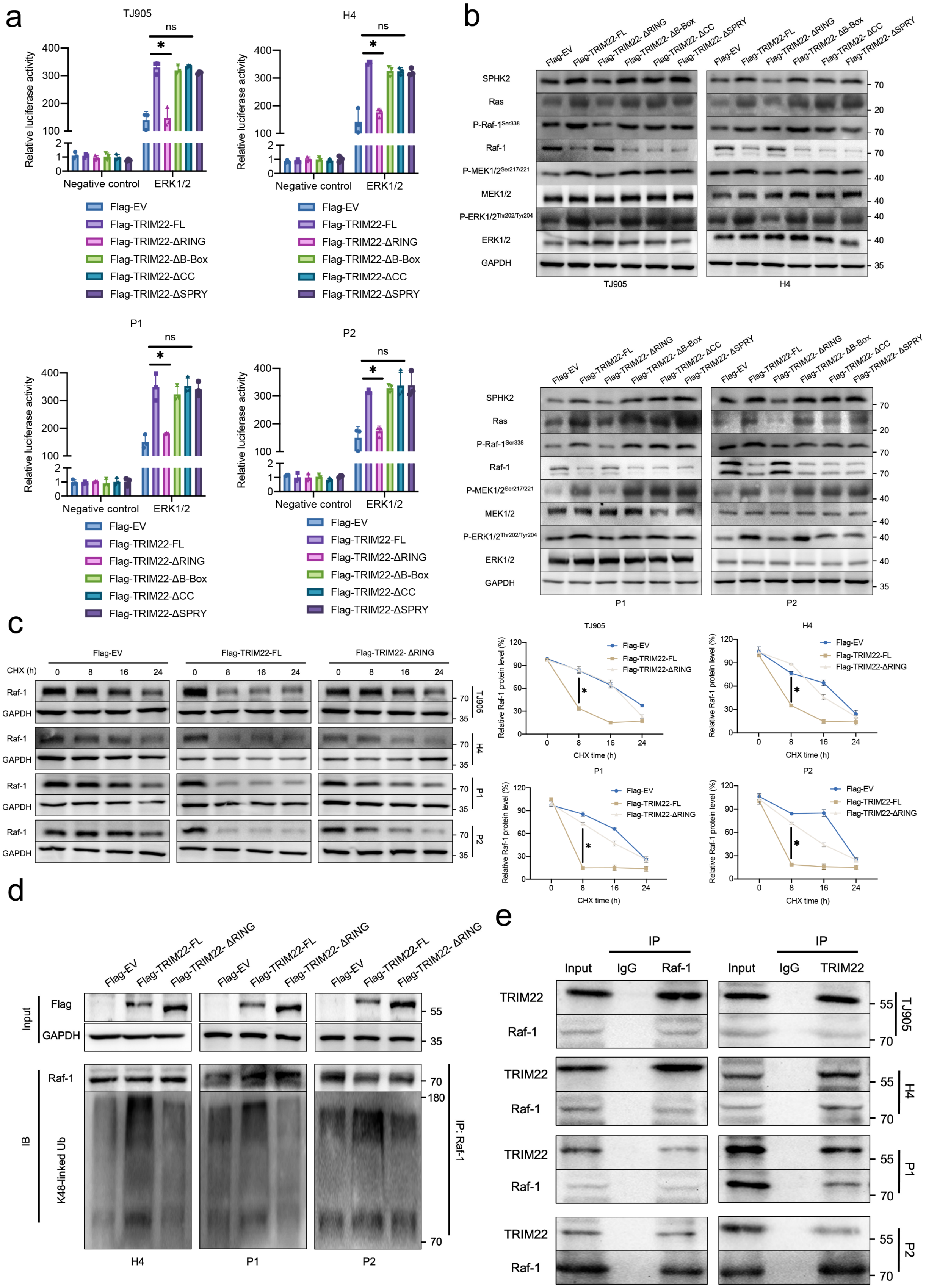
a
b
c
d
e
TJ905
H4
P1
P2
Relative luciferase activity
Negative control
ERK1/2
ns
*
Flag-EV
Flag-TRIM22-FL
Flag-TRIM22-ΔRING
Flag-TRIM22-ΔB-Box
Flag-TRIM22-ΔCC
Flag-TRIM22-ΔSPRY
SPHK2
Ras
P-Raf-1Ser338
Raf-1
P-MEK1/2Ser217/221
MEK1/2
P-ERK1/2Thr202/Tyr204
ERK1/2
GAPDH
CHX (h)
Relative Raf-1 protein level (%)
CHX time (h)
Input
IP
IgG
TRIM22
Flag
IB
K48-linked Ub
IP: Raf-1

Supplementary Fig. 7 TRIM22 binds to Raf-1 and regulates SPHK2/MAPK signaling through its RING domain.

a Luciferase activity from TJ905[$F_{interaction}(5, 24) = 52.87$, P<0.0001], H4[$F_{interaction}(5, 24) = 48.17$, P<0.0001], P1[$F_{interaction}(5, 24) = 28.60$, P<0.0001] and P2[$F_{interaction}(5, 24) = 20.34$, P<0.0001] transfected with different TRIM22 truncation mutants (n = 3 per group). **b** The effects of different TRIM22 truncation mutants on the core protein of SPHK2/MAPK pathway were detected by Western blot in TJ905, H4, P1 and P2. **c** Western blot analysis and quantification of Raf-1 protein in different modified cells treated with cycloheximide (CHX; 25 μg/mL) for 0, 8, 16, and 24 h(n = 3 per group) . TJ905: $F_{interaction}(6, 24) = 88.31$, P<0.0001; H4: $F_{interaction}(6, 24) = 52.60$, P<0.0001; P1: $F_{interaction}(6, 24) = 164.1$, P<0.0001; P2: $F_{interaction}(6, 24) = 102.3$, P<0.0001. **d** In vivo ubiquitination assay of Raf-1 in modified H4, P1 and P2. **e** exogenous binding of TRIM22 and Raf-1 in TJ905, H4, P1 and P2 using anti-TRIM22 and anti-Raf-1 antibodies. The data were analyzed using two-way ANOVA and all data are expressed as the mean ± standard deviation. *P < 0.05 represents a statistically significant difference between the two groups. ns, not significant. Each experiment was repeated three times.

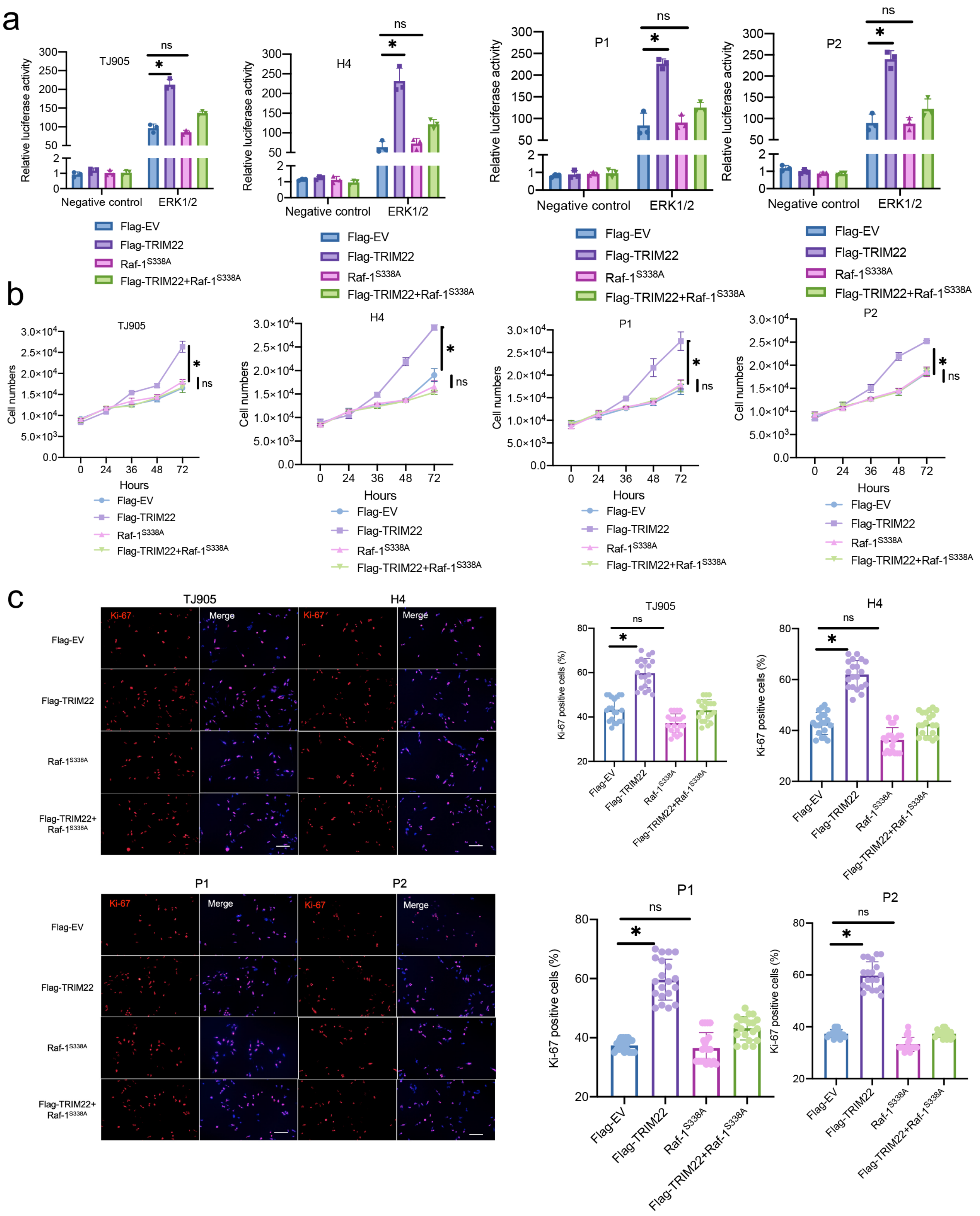
a
TJ905
H4
P1
P2
Relative luciferase activity
Negative control
ERK1/2
ns
*
Flag-EV
Flag-TRIM22
Raf-1S338A
Flag-TRIM22+Raf-1S338A
b
TJ905
H4
P1
P2
Cell numbers
Hours
ns
*
Flag-EV
Flag-TRIM22
Raf-1S338A
Flag-TRIM22+Raf-1S338A
c
TJ905
H4
P1
P2
Ki-67
Merge
Flag-EV
Flag-TRIM22
Raf-1S338A
Flag-TRIM22+Raf-1S338A
Ki-67 positive cells (%)
ns
*

Supplementary Fig. 8 Raf-1 regulates TRIM22-induced GBM proliferation in vivo.

a Luciferase activity from TJ905 [$F_{interaction}(3, 16) = 104.4$, $P<0.0001$], H4 [$F_{interaction}(3, 16) = 44.18$, $P<0.0001$], P1 [$F_{interaction}(3, 16) = 38.38$, $P<0.0001$] and P2 [$F_{interaction}(3, 16) = 39.58$, $P<0.0001$] transfected with Flag-TRIM22 and Raf-1^{S338A}, along with a reporter plasmid carrying the ERK1/2 promoter relative to negative control (n = 3 per group). **b** Growth curves generated using cell counting over 72 h for the cells indicated (n = 3 per group). TJ905: $F_{interaction}(12, 40) = 32.25$, $P<0.0001$; H4: $F_{interaction}(12, 40) = 66.84$, $P<0.0001$; P1: $F_{interaction}(12, 40) = 21.01$, $P<0.0001$; P2: $F_{interaction}(12, 40) = 27.40$, $P<0.0001$. **c** Representative images and quantification of Ki-67 immunofluorescence staining from modified TJ905 [$F (3, 76) = 68.35$, $P<0.0001$], H4 [$F (3, 76) = 107.7$, $P<0.0001$], P1 [$F (3, 76) = 96.78$, $P<0.0001$] and P2 [$F (3, 76) = 284.4$, $P<0.0001$] cells (n = 20 per group). Scale bar: 50 μm. The data were analyzed using one-way ANOVA (**c**) or two-way ANOVA (**a** and **b**) and all data are expressed as the mean ± standard deviation. *$P < 0.05$ represents a statistically significant difference between the two groups. ns, not significant. Each experiment was repeated three times.

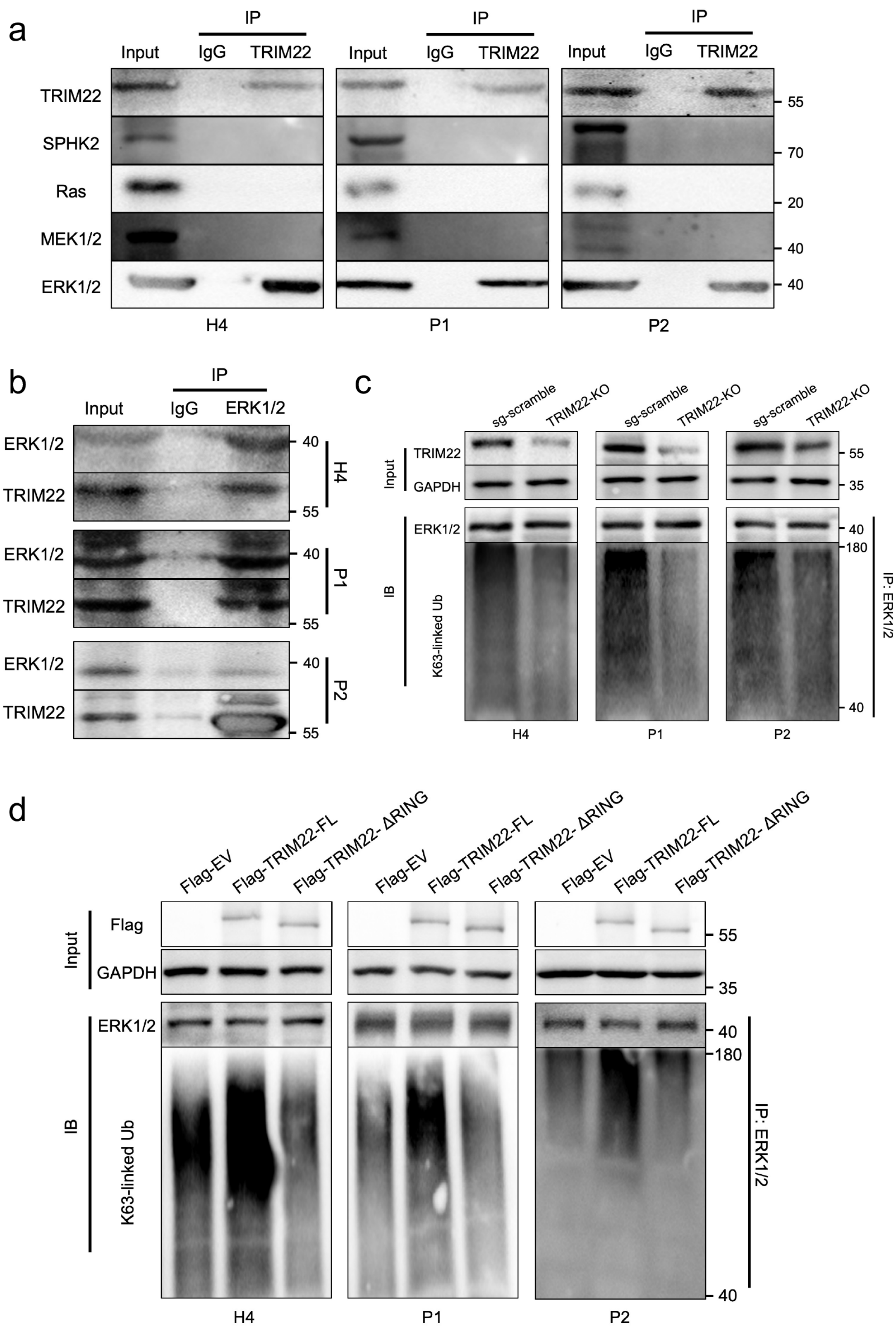
a
IP
Input
IgG
TRIM22
TRIM22
SPHK2
Ras
MEK1/2
ERK1/2
55
70
20
40
40
H4
P1
P2
b
IP
Input
IgG
ERK1/2
ERK1/2
TRIM22
H4
P1
P2
c
sg-scramble
TRIM22-KO
Input
GAPDH
IB
K63-linked Ub
IP: ERK1/2
180
35
d
Flag-EV
Flag-TRIM22-FL
Flag-TRIM22- ΔRING
Flag

Supplementary Fig. 9 TRIM22 promotes K63-linked ubiquitination of ERK1/2 through its RING domain.

a Anti-TRIM22 antibody was used to detect the binding of TRIM22 to SPHK2/MAPK pathway core protein in H4, P1 and P2. **b** Association of TRIM22 with ERK1/2 in H4, P1 and P2. **c-d** Western blot analysis for ERK1/2 of Co-IPs with K63-linkage specific polyubiquitin antibody in the indicated modified H4, P1 and P2 cells. Each experiment was repeated three times.

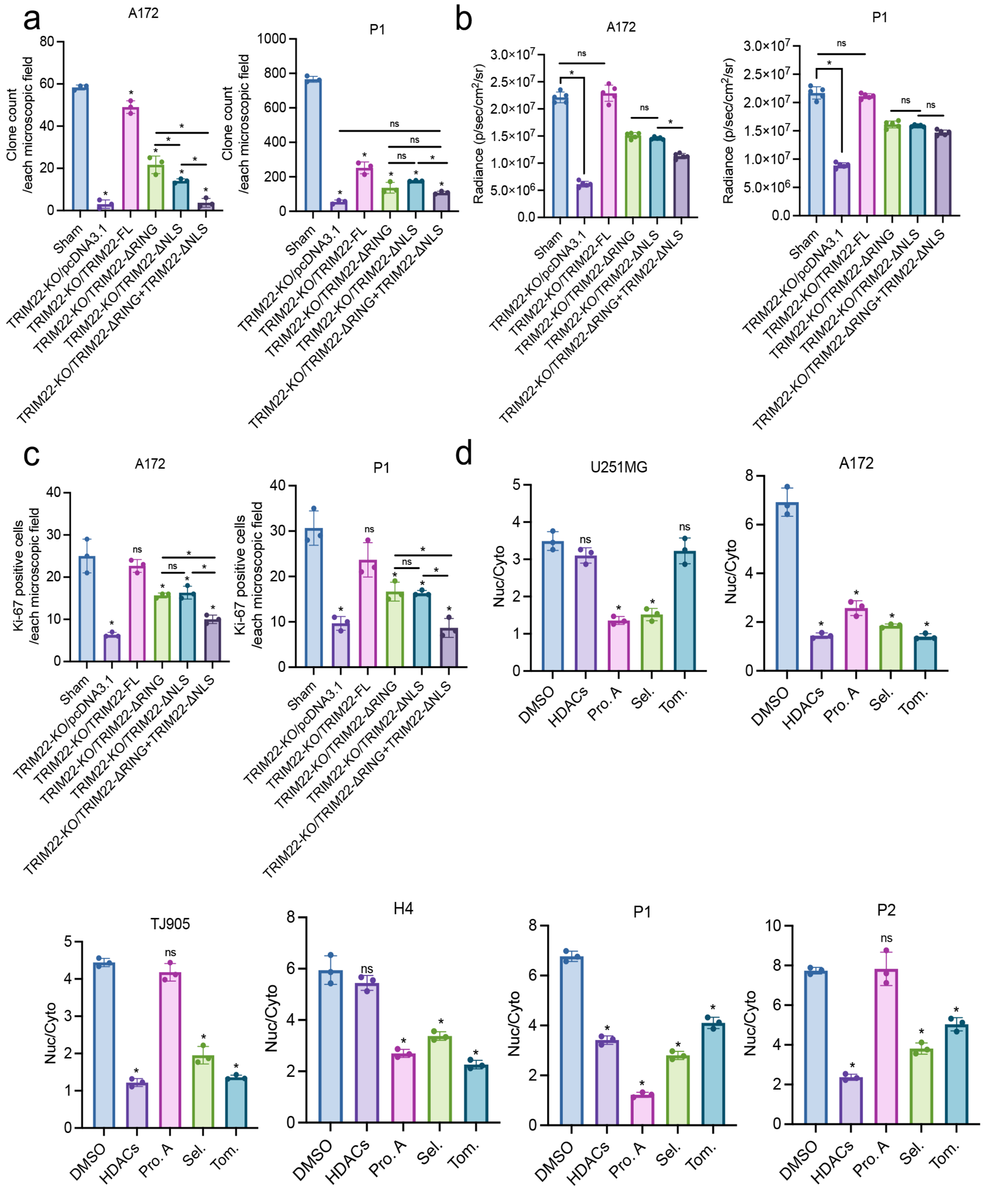
a
A172
Clone count
/each microscopic field
P1
b
Radiance (p/sec/cm²/sr)
c
Ki-67 positive cells
/each microscopic field
d
U251MG
Nuc/Cyto
TJ905
H4
P2
Sham
TRIM22-KO/pcDNA3.1
TRIM22-KO/TRIM22-FL
TRIM22-KO/TRIM22-ΔRING
TRIM22-KO/TRIM22-ΔNLS
TRIM22-KO/TRIM22-ΔRING+TRIM22-ΔNLS
DMSO
HDACs
Pro. A
Sel.
Tom.
ns
*

Supplementary Fig. 10 RING domain and NLS are important structures that TRIM22 promotes GBM proliferation.

a quantification of result in figure 8 panel a (n = 3 per group). A172: $F(5, 12) = 268.4$, $P<0.0001$; P1: $F(5, 12) = 470.2$, $P<0.0001$. **b** quantification of result in figure 8 panel b (n = 5 mice per group). A172: $F(5, 24) = 304.9$, $P<0.0001$; P1: $F(5, 24) = 297.0$, $P<0.0001$. **c** quantification of result in figure 8 panel c (n = 3 per group). A172: $F(5, 12) = 41.42$, $P<0.0001$; P1: $F(5, 12) = 31.75$, $P<0.0001$. **d** quantification of result in figure 8 panel e (n = 3 per group). U251MG: $F(4, 10) = 58.69$, $P<0.0001$; A172: $F(4, 10) = 178.2$, $P<0.0001$; TJ905: $F(4, 10) = 264.0$, $P<0.0001$; H4: $F(4, 10) = 86.07$, $P<0.0001$; P1: $F(4, 10) = 396.4$, $P<0.0001$; P2: $F(4, 10) = 91.06$, $P<0.0001$. The data were analyzed using one-way ANOVA and all data are expressed as the mean ± standard deviation. $*P < 0.05$ represents a statistically significant difference between the two groups or vs. control group. ns, not significant. Each experiment was repeated three times.

Supplementary Table 1 Sequences of sgRNAs

	Sequences
sg-TRIM22-1	ACGATCCCCTCAACCTCCGC
sg-TRIM22-2	TCAGACAAGAGAGAACCGCC
sg-TRIM22-3	CCAAACATTCCGCATAAACG

Supplementary Table 2 Primer sequence for ChIP-qPCR

Primers	Sequence
MAPKAPK3-F	5'-CCTGACACCCAACAGTAGCC-3'
MAPKAPK3-R	5'-TCTCCAGCAAGTTCCTTCCTC-3'
MAPKAPK3-1-F	5'-TCCACTGTCCCTTACCTCAAGTC-3'
MAPKAPK3-1-R	5'-CCACAATCGTCTCACAACCCT-3'
MAPKAPK3-2-F	5'-AAGTTGAGTACGCTGAGGGTTG-3'
MAPKAPK3-2-R	5'-CCTTGAGTCGGGTGTTTGCT-3'
MAPKAPK3-3-F	5'-TCTGGGCGGGACTCACTCTT-3'
MAPKAPK3-3-R	5'-CTGCTCCTCTGCTGTTTCACC-3'
NFATC2-F	5'-AAACAGGAGGAAACAGTGAGGG-3'
NFATC2-R	-5'-GGCTTACTCCAGAGGCAACG-3'
PPP3CC-F	5'-TGGAAAGTGAGGAGGGAGCAG-3'
PPP3CC-R	5'-TGTGGGTCATTCAACAAACGC-3'
PRKCA-F	5'-ATGCCCGCTACTCTTTCACA-3'
PRKCA-R	5'-CCACCAGAGCGACGATGTT-3'
SPHK1-F	5'-CTCAAGGCTGGTGGTAGTGG-3'
SPHK1-R	5'-CACTGTGAGTTCGTGGTGATAAA-3'
SPHK1-1-F	5'-AGGACCCATCATTCCGACAC-3'
SPHK1-1-R	5'-AGAAACAGGAACGAGGGGAG-3'
SPHK1-2-F	5'-CTACTGGCCTCCAAAGAAGTGA-3'
SPHK1-2-R	5'-GAGGCTTGACAGTGTAGATGGG-3'
SPHK1-3-F	5'-CTGCGAAGTTGAGCGAAAAG-3'
SPHK1-3-R	5'-TACCCAGTCGGTCCGGTTT-3'
SPHK2-F	5'-CTGGGAGCGAGATGCGTGTA-3'
SPHK2-R	5'-AGCGGTGAACCTGATAAGGAAAC-3'
SPHK2-1-F	5'-TGGAGACCCAGGGAGAAGTAG-3'
SPHK2-1-R	5'-AACTAGCAGAGCCAGGATTTGA-3'

SPHK2-2-F	5’-GACCTGACTCCTTGCTCCTACC-3’
SPHK2-2-R	5’-GTCCTGCTGCTCCTCTGCTT-3’
SPHK2-3-F	5’-CGTTATCATCACCCAACCACC-3’
SPHK2-3-R	5’-CCCCAGTAAGTATTCAGTACCCTTT-3’
SPHK2-4-F	5’-CAGAGCCACTCGCACCTTC-3’
SPHK2-4-R	5’-GCAGTCCTCGGAGCAGACA-3’

Supplementary Table 3 Position of SPHK2 primer for ChIP-qPCR

Sequence name	Position	Length (bp)
SPHK2	chr19: 49,122,548-49,133,974	11427
SPHK2/NM_001243876	chr19:49,122,763-49,133,662	10900
SPHK2/NM_001204160	chr19:49,128,286-49,133,662	5377
Peak	chr19:49,129,228-49,130,133	906
SPHK2-1 qPCR product	chr19:49,122,690-49,122,888	199
SPHK2-2 qPCR product	chr19:49,123,171-49,123,261	91
SPHK2-3 qPCR product	chr19:49,123,685-49,123,807	123
SPHK2-4 qPCR product	chr19:49,128,764-49,128,917	154
SPHK2-5 qPCR product	chr19:49,129,495-49,129,601	107

Supplementary Table 4 Details of the antibodies used in the experiment

Name	Article number	Company	Country
Anti-TRIM22	ab224059	Abcam	UK
Anti-GAPDH	ab59164	Abcam	UK
Anti-Histone H3	ab1791	Abcam	UK
Anti-DDDDK tag	ab205606	Abcam	UK
Anti-HA tag	ab9110	Abcam	UK
Anti-RNA polymerase II	GTX102535	GeneTex	USA
Anti-SPHK2	17096	Proteintech	China
Anti-Ras	67648	CST	USA
Anti-Phospho-c-Raf (Ser338)	9427	CST	USA
Anti-Raf-1	66592	Proteintech	China
Anti-Phospho-MEK1/2 (Ser217/221)	3958	CST	USA
Anti- MEK1/2	4694	CST	USA
Anti-Phospho-ERK1/2 (Thr202/Tyr204)	28733	Proteintech	China
Anti-ERK1/2	11257	Proteintech	China
Anti-Ki67	ab16667	Abcam	UK

Anti-Ubiquitin (linkage specific K48)	ab140601	Abcam	UK
Anti-Ubiquitin (linkage specific K63)	ab179434	Abcam	UK
K145	HY-15779	MCE	USA
HDACs	HY-114414	MCE	USA
Protosappanin A	HY-113573	MCE	USA
Selumetinib	HY-50706	MCE	USA
Tomatidine	HY-N2149	MCE	USA
